# Endogenous retroviral insertions drive non-canonical imprinting in extra-embryonic tissues

**DOI:** 10.1186/s13059-019-1833-x

**Published:** 2019-10-29

**Authors:** Courtney W. Hanna, Raquel Pérez-Palacios, Lenka Gahurova, Michael Schubert, Felix Krueger, Laura Biggins, Simon Andrews, Maria Colomé-Tatché, Deborah Bourc’his, Wendy Dean, Gavin Kelsey

**Affiliations:** 1 Epigenetics Programme, Babraham Institute, Cambridge, UK; 2 Centre for Trophoblast Research, University of Cambridge, Cambridge, UK; 3 Institut Curie, PSL University, Inserm, CNRS, Paris, France; 4 University of South Bohemia, Ceske Budejovice, Czech Republic; 5 Institute of Animal Physiology and Genetics, ASCR, Libechov, Czech Republic; 6 European Research Institute for the Biology of Ageing, University Medical Center Groningen, University of Groningen, Groningen, The Netherlands; 7 Bioinformatics, Babraham Institute, Cambridge, UK; 8 Helmholtz Zentrum München, German Research Center for Environmental Health, Institute of Computational Biology, Neuherberg, Germany; 9 TUM School of Life Sciences Weihenstephan, Technical University of Munich, Freising, Germany; 10 Present Address: Department of Biochemistry and Molecular Biology, University of Calgary, Calgary, Canada

**Keywords:** Genomic imprinting, Histone modifications, Extra-embryonic, Development, Embryo, H3K27me3, Non-canonical imprinting, Long terminal repeats (LTRs), Placenta, Endogenous retroviruses (ERVs)

## Abstract

**Background:**

Genomic imprinting is an epigenetic phenomenon that allows a subset of genes to be expressed mono-allelically based on the parent of origin and is typically regulated by differential DNA methylation inherited from gametes. Imprinting is pervasive in murine extra-embryonic lineages, and uniquely, the imprinting of several genes has been found to be conferred non-canonically through maternally inherited repressive histone modification H3K27me3. However, the underlying regulatory mechanisms of non-canonical imprinting in post-implantation development remain unexplored.

**Results:**

We identify imprinted regions in post-implantation epiblast and extra-embryonic ectoderm (ExE) by assaying allelic histone modifications (H3K4me3, H3K36me3, H3K27me3), gene expression, and DNA methylation in reciprocal C57BL/6 and CAST hybrid embryos. We distinguish loci with DNA methylation-dependent (canonical) and independent (non-canonical) imprinting by assaying hybrid embryos with ablated maternally inherited DNA methylation. We find that non-canonical imprints are localized to endogenous retrovirus-K (ERVK) long terminal repeats (LTRs), which act as imprinted promoters specifically in extra-embryonic lineages. Transcribed ERVK LTRs are CpG-rich and located in close proximity to gene promoters, and imprinting status is determined by their epigenetic patterning in the oocyte. Finally, we show that oocyte-derived H3K27me3 associated with non-canonical imprints is not maintained beyond pre-implantation development at these elements and is replaced by secondary imprinted DNA methylation on the maternal allele in post-implantation ExE, while being completely silenced by bi-allelic DNA methylation in the epiblast.

**Conclusions:**

This study reveals distinct epigenetic mechanisms regulating non-canonical imprinted gene expression between embryonic and extra-embryonic development and identifies an integral role for ERVK LTR repetitive elements.

## Background

The genetic contributions from both the sperm and oocyte are essential for successful development in mammals. Thirty-five years ago, seminal embryo manipulation experiments in mice showed that embryos with either two maternal or two paternal genomes die early in gestation [1, 2], and it was postulated that the parental genomes were somehow differentially imprinted during gametogenesis. Shortly thereafter, three genes, *Igf2r*, *H19*, and *Igf2*, were identified to be expressed mono-allelically based on the parent of origin, revealing the first examples of “genomic imprinting” [3–5]. Importantly, the regulation of imprinted mono-allelic expression was found to be due to the asymmetric deposition of an epigenetic mark, DNA methylation, in gametes [6, 7].

The study of imprinted genes has been integral to our understanding of epigenetic regulation of gene expression and has revealed the capacity for intergenerational transmission of epigenetic instructions from gametes to a newly formed embryo. Imprinting classically depends on locus-specific differences in DNA methylation established in the gametes [8, 9], with the vast majority of germ line differentially methylated regions (gDMRs) being established on maternal alleles during oogenesis [10]. Upon fertilization, despite the widespread epigenetic reprogramming, which includes the erasure of DNA methylation, reallocation of histone modification patterns, and dynamic chromatin remodeling [11], imprinted gDMRs are protected from these reprogramming events. In the post-implantation embryo, as there is re-acquisition of genomic DNA methylation, gDMRs maintain their inherited mono-allelic status through the protection of the unmethylated allele [12].

Imprinted genes are essential for the regulation of mammalian development, placentation, and fetal growth. It has been proposed that imprinting arose as a consequence of the conflict between the paternal and maternal genomes within the conceptus in placental mammals to increase or restrict demand for maternal resources, respectively [13]. The barrier between the mother and fetus, the extra-embryonic tissues, perhaps unsurprisingly, has more expressed imprinted genes than most other tissues [14, 15]. Furthermore, several observations suggest that imprinted gene regulation in extra-embryonic tissues may be dependent on a unique combination of multiple epigenetic layers, utilizing differential DNA methylation together with or in addition to histone modifications and long non-coding RNAs (lncRNAs) [16, 17].

Histone modifications H3K27me3 and H3K9me2/3 have been associated with placental-specific imprinting of distal genes in the *Kcnq1/Kcnq1ot1* and *Igf2r/Airn* clusters; however, this distal mono-allelic silencing is mediated by a non-coding RNA that is regulated by a canonical gDMR [16, 18]. Intriguingly, a number of isolated placental-specific imprinted genes (e.g., *Sfmbt2*, *Zfp64*, *Phf17*, *Smoc1*, *Pde10a*) appear to have no associated gDMRs, suggesting they may be solely regulated by histone modifications [15, 19, 20]. Indeed, a recent study found that maternally deposited H3K27me3 can confer imprinted gene expression. However, this “non-canonical” imprinting appears to be predominantly transient in the early embryo, and the key mechanisms that maintain this form of imprinting are still unknown [21]. Notably, for the few genes with persistent mono-allelic expression in later development, mono-allelic expression becomes restricted to extra-embryonic lineages, suggesting that extra-embryonic tissues may be uniquely permissive for this additional form of imprinted gene regulation. Importantly, it remains to be shown whether (1) histone modifications have a unique allelic patterning in extra-embryonic tissues conferring imprinted gene expression and (2) non-canonical imprinting is truly independent of maternally inherited gDMRs.

## Results

### Study design

**Fig. 1:**
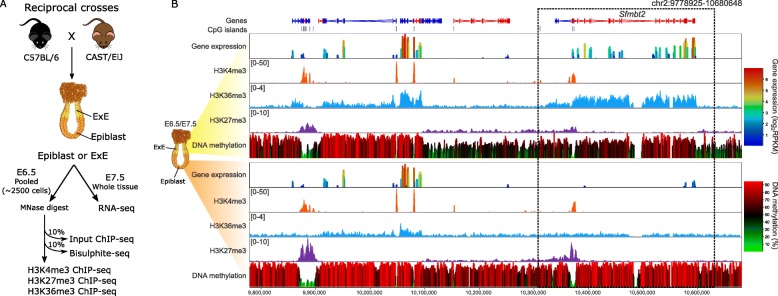
Experimental design and data evaluation. **a** Schematic of experimental design demonstrating the collection of reciprocal hybrid post-implantation embryos for ultra low-input ChIP-seq, bisulphite-seq, and RNA-seq. Two replicates of H3K4me3, H3K27me3, and H3K36me3 ChIP-seq were each done using a pool of either E6.5 epiblasts (*N* = 4) or ExE (*N* = 8), approximating an input of ~ 2500 cells. Two 10% inputs were taken from each pool of embryos, one for a ChIP-seq input control and the other for low-coverage bisulphite-seq. RNA-seq was done on matched single E7.5 epiblast (*N* = 3) and ExE (*N* = 3). **b** Screenshot of E7.5 gene expression; E6.5 H3K4me3, H3K36me3, and H3K27me3; and E6.5 DNA methylation for B6/CAST epiblast and ExE. H3K4me3 is enriched at gene promoters, H3K36me3 along gene bodies of expressed genes, and H3K27me3 at transcriptionally silent promoters. The epiblast is highly methylated with exception of promoters, while ExE shows the expected lower global levels of DNA methylation. The box highlights the *Sfmbt2* gene, which shows tissue-specific expression in ExE. ChIP-seq enrichment (RPKM) is shown for 1-kb running windows, with a 100-bp step (scales in square brackets), while gene expression and DNA methylation are shown using 2-kb running windows, with a 500-bp step

To evaluate the allelic regulation of gene expression in the embryo, we assessed the epigenetic modifications in C57BL6/Babr and CAST/EiJ reciprocal hybrid (denoted as B6/CAST and CAST/B6, in which by convention, the maternal strain is indicated first) embryonic day (E) 6.5 epiblast and extra-embryonic ectoderm (ExE). We assayed H3K4me3, H3K36me3, and H3K27me3 using ultra low-input ChIP-seq and DNA methylation using post-bisulphite adaptor tagging (PBAT), as previously described [22], from a pool of ~ 2500 cells of either epiblast or ExE (Fig. 1a, b; Additional file 1: Figure S1 and S2). We additionally profiled these epigenetic marks in E6.5 hybrid embryos derived from B6 females with a double conditional knockout for *Dnmt3a* and *Dnmt3b* in oocytes, driven by Zp3-cre, crossed to CAST males (denoted matDKO/CAST) (Additional file 1: Figure S1 and S2). Consequently, these matDKO/CAST embryos will inherit no maternal DNA methylation [9] but are able to sufficiently establish DNA methylation post-fertilization [23]. Allelic gene expression was evaluated in E7.5 epiblast and ExE of all hybrid crosses (Fig. 1a, b; Additional file 1: Figure S3). Details of biological replicates and datasets generated for this study are summarized in Additional file 2: Table S1.

### Imprinted H3K4me3 is associated with imprinted gene expression

**Fig. 2:**
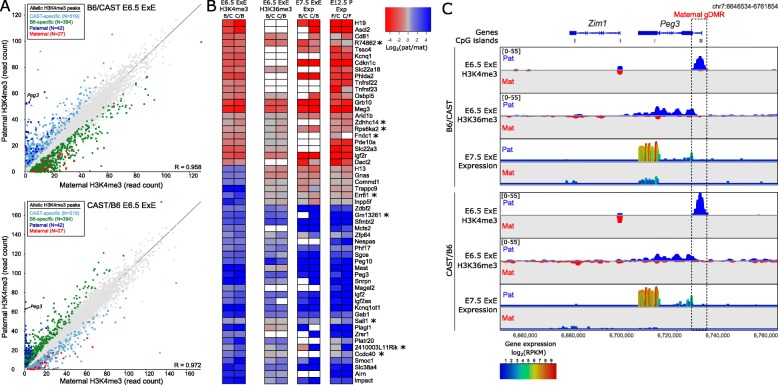
Imprinted H3K4me3 peaks are associated with imprinted gene expression in ExE. **a** Scatter plots of allelic H3K4me3 enrichment at autosomal H3K4me3 peaks (*N* = 15,976) in B6/CAST E6.5 ExE (top) and CAST/B6 E6.5 ExE (bottom). Peaks with allelically biased H3K4me3 were identified using EdgeR statistic (*p* < 0.05, corrected for multiple comparisons). Significant peaks were then classified into strain-specific allelic H3K4me3 if their allelic enrichment switched in the reciprocal cross, denoted as B6-specific (green) and CAST-specific (turquoise). Significant peaks were identified as imprinted if the allelic enrichment was consistent between reciprocal crosses, denoted as paternal (blue) or maternal (red). Enrichment is quantitated as read count normalized to library size, correcting for peak length. **b** Heatmap showing allelic bias (log_2_(pat/mat)) for E6.5 ExE H3K4me3 at H3K4me3 peaks identified in E6.5 ExE. Allelic bias (log_2_(pat/mat)) for E6.5 ExE H3K36me3, E7.5 ExE gene expression, and E12.5 placenta (P) gene expression is shown for associated nearby genes (Additional file 2: Table S2). Reciprocal hybrids are denoted as B/C (B6/CAST), C/B (CAST/B6), F/C (FvB/CAST), and C/F (CAST/FvB). White boxes indicate where there was insufficient data (ChIP-seq < 20 SNP-spanning reads in all replicates, RNA-seq < 5 SNP-spanning reads in all replicates). ChIP-seq data was quantitated is as in **a**, RNA-seq data was quantitated as read count over exons. H3K4me3 peaks were excluded if there was no gene within 10 kb or the associated gene was uninformative in all datasets. H3K4me3 peaks overlapping more than one gene promoter are duplicated in the H3K4me3 column. Novel imprinted genes are marked with an asterisk. **c** Screenshot of allelic enrichment for H3K4me3 and H3K36me3 in E6.5 ExE and gene expression in E7.5 ExE for B6/CAST and CAST/B6 at the known imprinted gene *Peg3*. Box indicates the location of the maternal gDMR. ChIP-seq data is quantitated using enrichment normalized RPKM for autosomal 1-kb running windows with a 100-bp step (scales in square brackets); paternal (blue) and maternal (red) enrichments are shown on mirrored axes. Gene expression is quantitated as log_2_(RPKM) for 500-bp running windows with a 50-bp step

To identify imprinted domains in E6.5 embryos, we called H3K4me3 peaks on autosomes in the epiblast (*N* = 33,329) and ExE (*N* = 40,468) of B6/CAST and CAST/B6 embryos. H3K4me3 peaks with a minimum of 20 strain-specific SNP-spanning reads in at least 1 replicate of epiblast (*N* = 15,407) and ExE (*N* = 15,976) were evaluated for allelic bias using EdgeR (*p* < 0.05, corrected for multiple comparisons). A consensus set of allelic H3K4me3 peaks was identified for epiblast (*N* = 329) and ExE (*N* = 913), as those that were significant in both B6/CAST and CAST/B6 crosses (Fig. 2a; Additional file 1: Figure S4). The vast majority of allelic H3K4me3 peaks demonstrated strain-specific inheritance patterns (92%), with the remaining 8% of peaks showing parent-of-origin (imprinted) inheritance. In total, we identified 69 imprinted H3K4me3 peaks in ExE and 29 in the epiblast (Fig. 2a; Additional file 1: Figure S4; Additional file 2: Tables S2 and S3). The majority (72.4%) of imprinted H3K4me3 peaks were located at an annotated gene promoter(s), and the remaining were assigned to the nearest gene within 10 kb, where applicable (Additional file 2: Tables S2 and S3). When compared to a list of known (and putative) imprinted genes (Additional file 2: Table S4), known imprinted genes comprised 77.8% and 96.2% of genes associated with an imprinted H3K4me3 peak in ExE and epiblast, respectively.

We then evaluated whether imprinted H3K4me3 was associated with allele-specific gene expression, using the EdgeR statistical approach to identify genes with allelic bias for H3K36me3 in E6.5 epiblast and ExE, and gene expression in E7.5 epiblast, E7.5 ExE, and E12.5 placenta [15] (Fig. 2b; Additional file 1: Figure S4). In epiblast, 85.2% (23/27) of informative genes associated with an imprinted H3K4me3 peak (Additional file 1: Figure S4) showed a significant allelic bias in at least one dataset (Additional file 2: Table S3). In ExE, 94.4% (51/54) of informative genes associated with an imprinted H3K4me3 peak (Fig. 2b) showed a significant allelic bias in at least one dataset (Additional file 2: Table S2). Thus, imprinted H3K4me3 peaks are strongly predictive of mono-allelic H3K36me3 and gene expression of the nearest genes, as demonstrated by the *Peg3* gene (Fig. 2c; Additional file 1: Figure S4).

### Non-canonical vs. canonical imprinted gene regulation

**Fig. 3:**
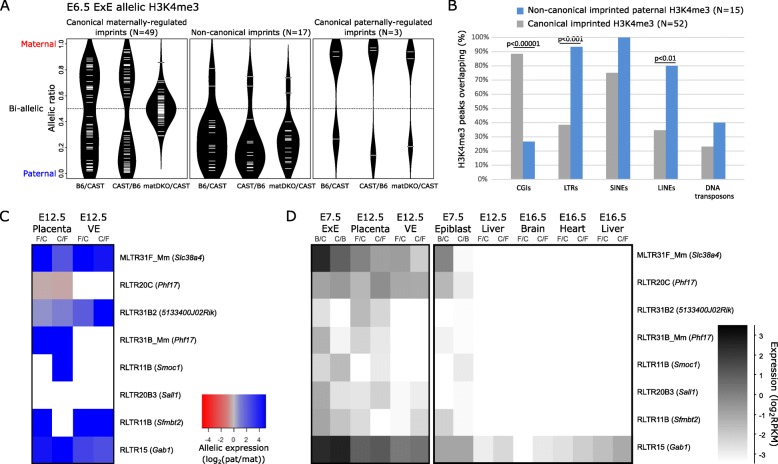
Non-canonical imprinted H3K4me3 in ExE demarcates imprinted ERVK LTR elements with extra-embryonic-specific imprinted expression. **a** Allelic ratio for H3K4me3 at canonical maternally regulated imprinted H3K4me3 peaks (*N* = 49) canonical paternally regulated imprinted H3K4me3 peaks (*N* = 3), and non-canonical imprinted H3K4me3 peaks (*N* = 17) in B6/CAST, CAST/B6, and matDKO/CAST E6.5 ExE. Informative H3K4me3 peaks were quantitated using read counts corrected for library size, and relative allelic ratios were calculated (allelic ratio = mat/(mat + pat)). **b** The percentage of non-canonical imprinted H3K4me3 peaks with paternal allelic bias (*N* = 15) and canonical imprinted H3K4me3 peaks (*N* = 52) that were overlapping each category of genomic feature, including CpG islands (CGIs) and classes of repetitive elements. Each pair-wise comparison was done using chi-square statistic, with a significance threshold adjusted for multiple comparisons using Bonferroni correction. **c** Allelic expression of transcribed ERVK LTRs within a non-canonical imprinted paternal H3K4me3 peak (*N* = 8, Additional file 2: Table S5) is shown in extra-embryonic tissues at E12.5 (placenta and visceral endoderm (VE)). Reciprocal hybrids are denoted as F/C (FvB/CAST) and C/F (CAST/FvB). White boxes indicate where there was insufficient data (< 5 SNP-spanning reads in all replicates). **d** Heatmap showing expression levels across extra-embryonic and embryonic tissues of transcriptionally active ERVK LTR elements within a non-canonical imprinted paternal H3K4me3 peak (*N* = 8). The nearest gene is denoted in brackets next to the ERVK LTR identifier

To determine which imprinted loci are dependent on maternally inherited gDMRs, we evaluated allelic H3K4me3 in post-implantation matDKO/CAST embryos. Using the EdgeR statistical approach described for the reciprocal hybrids, we identified H3K4me3 peaks that lost allelic bias in the matDKO/CAST (canonical maternal imprints) and those that remained imprinted (non-canonical imprints and canonical paternal imprints) (Fig. 3a; Additional file 1: Figure S5). In epiblast, there were only 5 imprinted H3K4me3 peaks present in the matDKO/CAST (*H19*, IG-DMR, *Meg3*, *Slc38a4*, and *Gab1*); the former 3 are regulated by paternal gDMRs, thus leaving 2 that could be classified as non-canonical (Additional file 2: Table S3). In ExE, we identified 3 H3K4me3 peaks associated with known paternal gDMRs (*H19*, *Igf2*, and *Meg3*), 17 that we classified as non-canonical (including all 4 previously reported non-canonical imprinted genes [21]), with a remaining 49 canonical maternally regulated imprinted H3K4me3 peaks that were lost in matDKO/CAST (Fig. 3a; Additional file 2: Table S2). These data support previous reports that non-canonical imprinting is largely restricted to the extra-embryonic lineage [21].

Two non-canonical imprinted H3K4me3 peaks identified in ExE were on the maternal alleles (*Pde10a* and *Cd81*) and were localized at the large *Igf2r/Airn* and *Kcnq1/Kcnq1ot1* imprinted clusters, and thus have distinct regulatory mechanisms to non-canonical H3K4me3 peaks on paternal alleles [15]. Thus, all subsequent analyses have been done on the 15 non-canonical imprinted paternal H3K4me3 peaks identified in ExE.

### Non-canonical imprinted H3K4me3 peaks localize to endogenous retroviral LTRs

We evaluated whether canonical and non-canonical imprints in ExE are enriched for similar genomic features. While canonical imprinted H3K4me3 peaks were strongly enriched for CGIs (88%), non-canonical imprinted H3K4me3 peaks were enriched for regulatory sequences of repetitive elements, the most significant of which was the long terminal repeats (LTRs) of endogenous retroviral (ERV) (93%) (Fig. 3b), specifically endogenous retrovirus-K (ERVKs) (Additional file 1: Figure S5).

As ERV LTRs have been implicated in regulating tissue-specific gene expression [24], we identified those ERVK LTRs within non-canonical imprinted H3K4me3 peaks that were transcription initiation sites in extra-embryonic tissues (*N* = 8 out of 28) (Additional file 1: Figure S5; Additional file 2: Table S5). Similar to the genes associated with non-canonical imprinted H3K4me3 peaks (Fig. 2b) [21], ERVK LTR promoters underlying imprinted paternal H3K4me3 peaks showed predominantly imprinted paternal expression in E12.5 FvB x CAST hybrid placenta and visceral endoderm [15] (Fig. 3c). Similarly, using publically available RNA-seq data [15], we observed they were also expressed specifically in extra-embryonic lineages during post-implantation development (Fig. 3d).

### Genomic and epigenetic features associated with non-canonically imprinted ERVK LTRs

We then sought to determine (1) whether sequence or genomic features underlie the tissue specificity of extra-embryonic ERVK LTR promoters and (2) why a subset is non-canonically imprinted. To evaluate these questions, we identified all ERVK LTRs that fell within ExE H3K4me3 peaks that were active promoters in extra-embryonic tissues (*N* = 40), which included the 8 non-canonical imprinted ERVK LTRs and 32 ERVK LTRs without imprinted expression (Additional file 2: Table S6). Using these 40 extra-embryonic ERVK LTR promoters, we assessed the sequence composition, sequence motifs, proximity to genes and promoters, ERVK LTR classes, and LTR length.

**Fig. 4:**
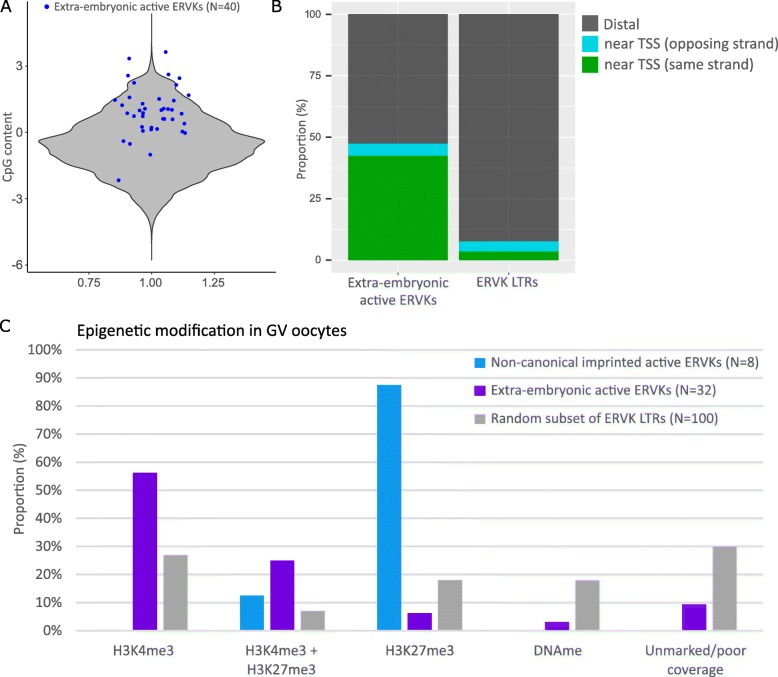
Genomic features and epigenetic patterning in the maternal oocyte are associated with ERVK LTR expression patterns in extra-embryonic tissues. **a** CpG content was compared between ERVK LTRs that were transcriptionally active in extra-embryonic tissues, including the subset of non-canonically imprinted ERVKs (*N* = 40, blue dots) and all mappable ERVK LTRs (*N* = 334,322) (*p* < 5E−10, Welch two-sample *t* test). **b** The proportion of transcriptionally active ERVK LTRs in extra-embryonic tissues (*N* = 40) within 3 kb of a transcription start site (TSS) on the same or opposing strand was compared to all mappable ERVK LTRs (*N* = 334,322) (chi-square statistic, *p* < 0.0001). **c** The proportion of transcriptionally active non-canonical imprinted ERVK LTRs (*N* = 8) and extra-embryonic active ERVK LTRs (*N* = 32) overlapping epigenetic modifications in GV oocytes was compared to a random subset of mappable ERVK LTRs (*N* = 100) (chi-square statistic, *p* = 0.0002 and *p* = 0.0001, respectively)

In contrast to ERVK LTRs genome-wide, we found that extra-embryonic ERVK LTR promoters had relatively high CpG content (Fig. 4a) and were more likely to be in close proximity and on the same strand as an annotated transcription start site (TSS) (Fig. 4b). Similar to the majority of ERVK LTRs in the genome, extra-embryonic ERVK LTR promoters were mostly solo LTR elements (417 ± 19 bp) (Additional file 1: Figure S6), which had lost their associated retroviral genes [24]. Solo LTRs that act as enhancers in extra-embryonic tissues have been reported to be enriched in transcription factor motifs ELF5, EOMES, and CDX2 [25]; however, we did not identify motifs that were enriched among extra-embryonic ERVK LTR promoters using an unbiased approach. Furthermore, we did not find significant enrichment specifically for ELF5, EOMES, or CDX2 motifs.

As non-canonical imprinting has been associated with maternal H3K27me3 inherited from the oocyte [21], we evaluated whether epigenetic marks (H3K4me3, H3K27me3, and DNA methylation) in the maternal oocyte were associated with the transcriptional status of ERVK LTR promoters in extra-embryonic tissues. Non-canonical imprinted ERVK LTR promoters were indeed significantly associated with oocyte H3K27me3 (*p* < 0.001) (Fig. 4c). Remarkably, H3K4me3 in the oocyte significantly differentiated those ERVK LTRs that were transcriptionally active in extra-embryonic tissues compared to inactive (*p* < 0.001) (Fig. 4c).

### Non-canonically imprinted ERVK LTR promoters mediate imprinting of nearby protein-coding genes

**Fig. 5:**
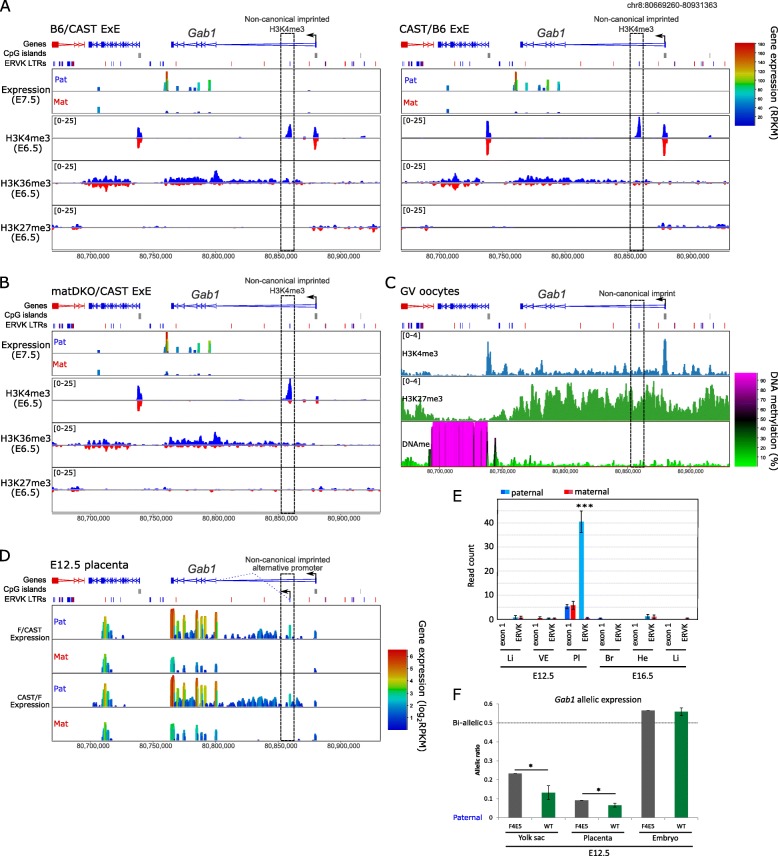
A non-canonically imprinted ERVK LTR drives imprinted expression of *Gab1* in placenta. **a** Screenshot of allelic gene expression, H3K4me3, H3K36me3, and H3K27me3 in B6/CAST and CAST/B6 ExE. ChIP-seq data is quantitated using enrichment normalized RPKM for 1-kb running windows with a 100-bp step (scales in square brackets); paternal (blue) and maternal (red) enrichments are shown on mirrored axes. RNA-seq data is quantitated as RPKM for 1-kb running windows with a 100-bp step. The box denotes the location of the non-canonical imprinted H3K4me3 peak associated with the known non-canonical imprinted gene *Gab1*. **b** Screenshot of allelic gene expression, H3K4me3, H3K36me3, and H3K27me3 in matDKO/CAST ExE, quantitated as in **a**. **c** Screenshot of H3K4me3, H3K27me3, and DNA methylation in GV oocytes. One-kilobase running windows with a 100-bp step were used; ChIP-seq data was quantitated as RPKM (scales in square brackets). **d** Screenshot showing allelic gene expression in F/CAST and CAST/F E12.5 placenta across the *Gab1* locus. The box depicts the non-canonical imprinted paternal H3K4me3 peak containing an imprinted transcriptionally active ERVK LTR element (RLTR15). RNA-seq data is quantitated as log_2_RPKM for 1000-bp running windows with a 100-bp step. **e** Read count for maternal (red) and paternal (blue) transcription is shown for the non-canonically imprinted RLTR15 and exon 1 of the *Gab1* gene in E12.5 and E16.5 embryonic (Li, liver; He, heart; Br, brain) and extra-embryonic (Pl, placenta; VE, visceral endoderm) tissues. Only intron-spanning reads were used, and two-tailed *t* test was used to statistically compare the allelic expression (***p < 0.0005). **f** Barplot shows the allelic gene expression (allelic ratio = mat/(mat + pat)) for the *Gab1* gene in B6/CAST E12.5 yolk sac, placenta, and whole embryos. F4E5 carried CRISPR-targeted deletion of non-canonically imprinted RLTR15 on the paternal allele and was compared to wild-type (WT) controls (*N* = 3). Two-tailed single sample *t* test was used to compare the F4E5 value to the WT mean (*p < 0.05). Error bars show standard deviation

We found examples of non-canonically imprinted ERVK LTR promoters driving transcription of non-coding RNAs, but also mediating imprinting of protein-coding genes. One such example is the non-canonically imprinted ERVK LTR (RLTR15) located in intron 1 of the *Gab1* gene. *Gab1* shows imprinted paternal expression in E7.5 ExE; yet, the promoter of *Gab1* has bi-allelic enrichment for H3K4me3 (Fig. 5a). Rather, the intronic RLTR15 is demarcated by imprinted paternal H3K4me3 (Fig. 5a) and is non-canonically imprinted (Fig. 5b) with enrichment for H3K27me3 in the oocyte (Fig. 5c). We find that RLTR15 acts as an alternative promoter for the *Gab1* gene on the paternal allele specifically in the placenta (Fig. 5d, e), with intron-spanning reads demonstrating that the ERVK LTR is spliced onto exon 2 (Additional file 1: Figure S7).

Together, these analyses suggest that ERVK LTR elements can directly mediate imprinted gene expression in extra-embryonic lineages. To demonstrate this using a genetic approach, we designed CRISPR/Cas9 sgRNAs to excise RLTR15 in vivo (Additional file 1: Figure S7). We targeted B6/CAST hybrid zygotes which were implanted in foster mothers, and we subsequently collected E12.5 extra-embryonic tissues (placenta and yolk sac) and whole embryos (*N* = 42 embryos). We were able to obtain one embryo (F4E5) that was targeted on the paternal CAST allele, although genotyping revealed that the deletion was mosaic (Additional file 1: Figure S7). Nevertheless, allelic RNA-seq analysis of F4E5 compared with three controls (Additional file 1: Figure S7) demonstrated that *Gab1* specifically showed a partial loss of imprinting in E12.5 placenta and yolk sac (Fig. 5f; Additional file 1: Figure S7).

Another intriguing example is imprinted gene *Slc38a4*. *Slc38a4* has a maternal gDMR at its promoter [26] but paradoxically was recently reported to have non-canonical imprinted gene expression in extra-embryonic lineages [21]. Furthermore, we identified a non-canonical imprinted H3K4me3 peak overlying the *Slc38a4* gDMR promoter (Additional file 2: Tables S2 and S3), raising questions as to whether the *Slc38a4* promoter is regulated canonically or non-canonically. To investigate this further, we assessed allelic RNA-seq patterns in the epiblast and ExE from B6/CAST, CAST/B6, and matDKO/CAST embryos in detail. B6/CAST and CAST/B6 epiblast and ExE showed the expected imprinted paternal expression (Additional file 1: Figure S8). In the matDKO/CAST epiblast, loss of the maternal DNA methylation at the gDMR resulted in bi-allelic expression (Additional file 1: Figure S8), consistent with canonical imprinting. However, in the matDKO/CAST ExE, while there was an increase in the expression of the maternal allele, there was still a twofold paternal bias in the expression (Additional file 1: Figure S8), an observation consistent with non-canonical imprinting.

In ExE, in addition to the non-canonical H3K4me3 peak at the annotated *Slc38a4* promoter, there were four upstream non-canonical H3K4me3 peaks, all of which were located over ERV LTR element insertions (Additional file 1: Figure S8). In particular, one ERVK LTR ~ 75 kb upstream (MLTR31F_Mm) was highly expressed in ExE and showed non-canonical imprinted expression of a spliced transcript from the paternal allele (Additional file 1: Figure S8). However, we found no evidence that this upstream ERVK LTR was acting an alternative promoter for *Slc38a4*, as there were no intron-spanning reads extending to the first or second exon of *Slc38a4* in E7.5 ExE or E12.5 placenta. Together, these data suggest that the annotated *Slc38a4* promoter is predominantly canonically imprinted by DNA methylation in embryonic lineages, while in extra-embryonic lineages, it appears that the non-canonically imprinted upstream ERVK LTRs may modulate the activity of the paternal allele of the *Slc38a4* promoter, resulting in non-canonical imprinted gene expression.

We evaluated publically available gene expression [27], DNA methylation [28], and H3K4me3 and H3K27me3 histone modifications [22] in GV oocytes to determine whether the germ line pattern of maternal epigenetic modifications across the *Slc38a4* locus is consistent with this finding. Indeed, the annotated promoter is fully methylated in GV oocytes, spanned by an oocyte-specific transcript emanating from multiple mammalian apparent LTR retrotransposon (MaLR) elements upstream (Additional file 1: Figure S8), as has been previously reported [27, 29]. In contrast, the upstream non-canonical imprinted H3K4me3 peaks are enriched for H3K27me3 in GV oocytes (Additional file 1: Figure S8). Thus, it appears that independent ERV LTR insertions upstream of the *Slc38a4* locus, one specifically active in oocytes and the other specifically active in extra-embryonic tissues, may have enabled genomic imprinting to have evolved twice at this locus, using both canonical and non-canonical mechanisms. While this finding needs to be confirmed genetically, it would represent, to our knowledge, the first such example of recurrent evolution of imprinting mechanisms reported to date.

### Epigenetic regulation of non-canonical imprints in post-implantation embryos

**Fig. 6:**
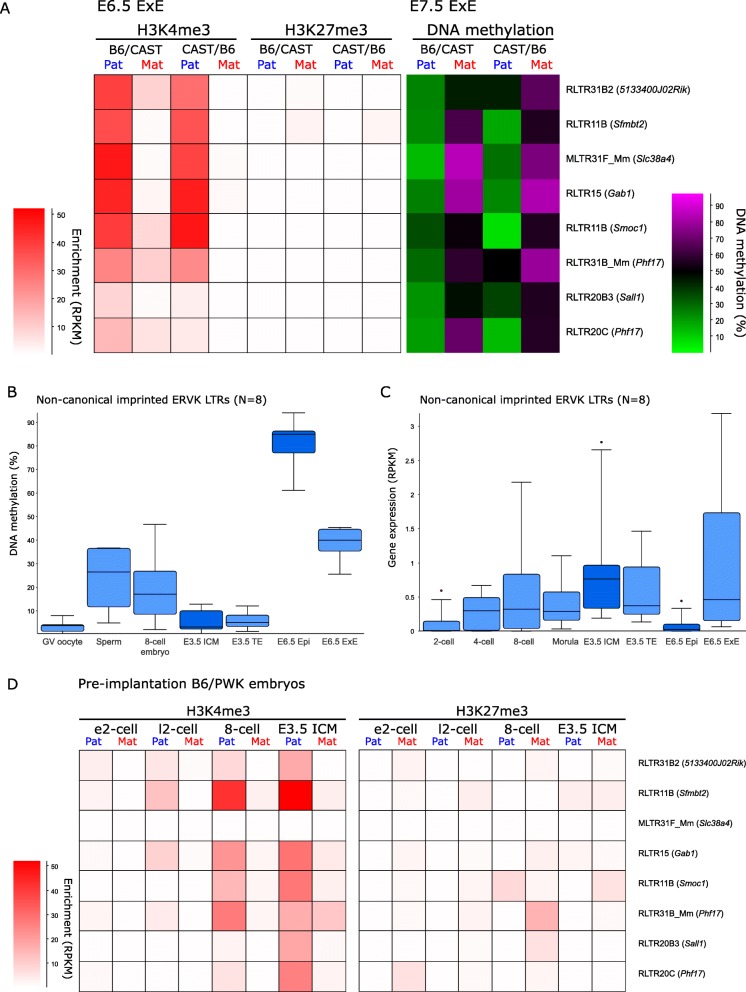
Non-canonically imprinted ERVK LTRs lose maternal H3K27me3 and acquire secondary imprinted DMRs in post-implantation ExE. **a** Heatmap showing enrichment for H3K4me3 and H3K27me3 and DNA methylation on the maternal and paternal allele in B6/CAST and CAST/B6 ExE at non-canonically imprinted active ERVK LTRs ± 500 bp (*N* = 8). DNA methylation of the maternal and paternal allele is shown in B6/CAST and CAST/B6 E7.5 ExE. **b** Boxplots show DNA methylation at non-canonically imprinted active ERVK LTRs ± 500 bp (*N* = 8) in germ cells and pre- and post-implantation stage C57BL/6 embryos. **c** Boxplots show gene expression for non-canonically imprinted active ERVK LTRs ± 500 bp (*N* = 8) in pre- and post-implantation embryonic stage C57BL6 embryos. **d** Heatmap showing enrichment for H3K4me3 and H3K27me3 on the maternal and paternal allele across pre-implantation development (e2-cell, early 2-cell embryo; l2-cell, late 2-cell embryo; 8-cell, 8-cell embryo; ICM, inner cell mass) in B6/PWK embryos at non-canonically imprinted active ERVK LTRs ± 500 bp (*N* = 8)

It has been shown that non-canonical imprinting in the early embryo is mediated by the inheritance of maternal H3K27me3 from the oocyte [21]. Therefore, we were surprised to find that non-canonical imprinted EVRK LTRs did not show enrichment for maternal H3K27me3 in E6.5 ExE (Figs. 5a and 6a). Although there was a subtle bias of H3K27me3 towards the maternal allele in ExE at non-canonical imprinted H3K4me3 peaks (*p* = 0.02), when we identified regions with imprinted H3K27me3 in ExE using the EdgeR statistical approach (Additional file 1: Figure S9), only one non-canonically imprinted H3K4me3 peak was associated with imprinted H3K27me3. Furthermore, we found that the vast majority of imprinted H3K27me3 in post-implantation ExE was localized to two large imprinting clusters (*Kcnq1/Kcnq1ot1* and *Igf2r/Airn*) and entirely dependent on maternal gDMRs (Additional file 1: Figure S9 and S10).

To determine whether another repressive epigenetic mark replaced maternal H3K27me3 in the post-implantation embryo, we assessed the allelic DNA methylation. We generated high coverage bisulphite sequencing data from ExE and epiblast of E7.5 reciprocal B6 x CAST hybrid embryos enabling us to obtain sufficient read depth over ERVK LTRs. These data revealed that non-canonical imprinted EVRK LTR promoters become DMRs in ExE, with the maternal allele becoming methylated (Fig. 6a), whereas both alleles were methylated in the epiblast (Additional file 1: Figure S11). Using publicly available bisulphite and RNA sequencing data from C57BL/6 germ cells and early embryos [30], we demonstrate that these regions are definitively tissue-specific secondary imprints acquired in the post-implantation de novo DNA methylation wave specifically in ExE (Fig. 6b). The acquisition of bi-allelic DNA methylation in the post-implantation epiblast corresponds to the silencing of these ERVK LTR promoters (Fig. 6c).

Conversely, using publically available ChIP-seq data [31], we observed the loss of maternal enrichment for H3K27me3 at non-canonical imprints during pre-implantation development (Fig. 6d; Additional file 1: Figure S11). Thus, non-canonical imprints do not maintain allelic H3K27me3 beyond early pre-implantation embryonic development, supporting that the regulation of allele-specific expression of non-canonical imprinted genes is superseded by DNA methylation in post-implantation development.

## Discussion

**Fig. 7:**
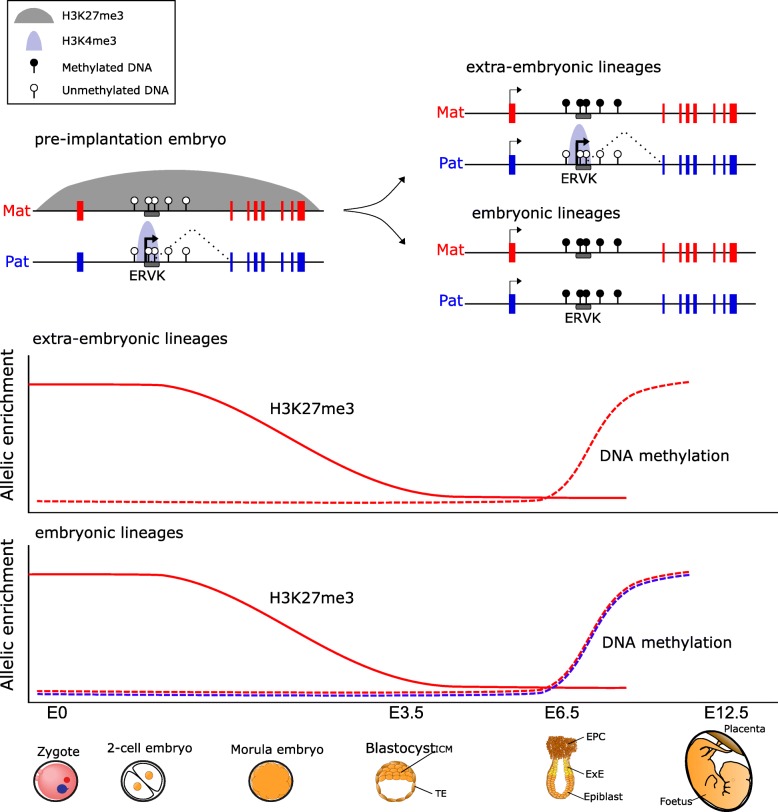
Summary of epigenetic regulation of non-canonical imprinting in embryonic development. Schematic diagram showing the allelic epigenetic regulation of a non-canonically imprinted gene by an ERVK LTR element (top) and the dynamic regulation of non-canonically imprinted ERVK LTRs across pre- and post-implantation development (bottom). In the pre-implantation embryo, inherited H3K27me3 from the oocyte silences the maternal allele. In the post-implantation embryo, maternal H3K27me3 transitions to imprinted maternal DNA methylation in extra-embryonic lineages, thereby retaining the imprinted paternal expression of the ERVK LTR. Alternatively, in the embryonic lineages, both the maternal and paternal alleles acquire DNA methylation, consequently silencing the ERVK LTR transcription. The maternal allele is shown in red, and paternal allele is shown in blue. In the allelic enrichment plot, the solid line is the level of H3K27me3, and the dashed line is the level of DNA methylation. Embryonic day (E) is shown on the *x*-axis for each respective stage of embryogenesis

In this study, we evaluated allelic histone modifications, DNA methylation, and gene expression to investigate the epigenetic regulation of imprinted genes in the post-implantation embryonic and extra-embryonic lineages. We identified non-canonical imprints that are definitively independent of maternally inherited DNA methylation in ExE and find that these are located preferentially at active ERVK LTR insertions. Furthermore, we find that while non-canonical imprinted genes inherit allelic H3K27me3 from the oocyte, this allelic enrichment is transient and their epigenetic regulation is superseded by secondary imprinted DMRs specifically acquired in extra-embryonic lineages (Fig. 7). Our findings not only reveal that non-canonical imprinting can be mediated by ERVK LTR insertions, but uncover the epigenetic mechanisms responsible for their persistence in extra-embryonic tissues.

The majority of the non-canonical imprinted H3K4me3 peaks we identified overlaid mono-allelically expressed ERVK LTR promoters, which mediated transcription of non-coding RNAs (e.g., *Platr20*, upstream of *Slc38a4*) or acted as alternative promoters to form chimeric mRNAs with nearby genes (e.g., *Gab1*, *Smoc1*). At the *Gab1* locus, we demonstrated that spliced transcripts from the ERVK LTR are exclusively expressed from the paternal allele, while the upstream canonical exon 1 is transcribed bi-allelically. Furthermore, when we genetically targeted the *Gab1* EVRK LTR promoter, despite only obtaining a mosaic deletion, we were able to disrupt the imprinted gene expression of *Gab1*. Together, these findings demonstrate that ERVK LTRs are a key genomic feature mediating non-canonical imprinting in murine extra-embryonic development.

ERVs have also been reported to function as enhancers specifically in the placenta through the acquisition of binding sites for developmental transcription factors, and it is thought that the uniquely hypomethylated state of the extra-embryonic tissues may enable transcriptional regulation by repetitive elements [25, 32]. We did not find any evidence for shared transcription factor binding motifs among active extra-embryonic ERVK LTR promoters; however, we found that they were predominantly CpG-rich solo LTRs. There are several epigenetic modifiers containing CxxC domains that bind unmethylated CpGs, such H3K4 methyltransferases [33]; thus, high CpG content may be key to their role in transcriptional regulation. Solo LTRs, in particular, may be co-opted as transcriptional regulators in development because their lack of viral genes may enable them to escape the KRAB-ZFP silencing [24]. Notably, ERVs genome-wide are under-represented within 5 kb of promoters and specifically in the sense orientation [34]. We find that extra-embryonic ERVK LTR promoters are not only in close proximity to TSSs, but in particular in the sense orientation. Together, these findings suggest that promoter activity of ERVK LTRs in extra-embryonic tissues may be attributable to sequence composition and opportunistic positioning in the genome.

Notably, ERV-derived placental enhancers and oocyte promoters were found to be species-specific [25, 29], and thus, we may expect that non-canonical imprinted regions, similarly co-opting ERV insertions, may also be species-specific. Indeed, preliminary studies in human embryos found five paternally expressed genes that may be regulated by maternal H3K27me3 [35], none of which have been reported to be imprinted in mice. These findings are reminiscent of placental-specific imprinted gDMRs in humans, which were also found to be species-specific [36, 37]. Furthermore, a recent study demonstrated that species-specific gDMRs were a consequence of unique ERV insertions, which initiated gDMR-spanning transcription in oocytes [29]. Together, these findings support that ERV activity in the placenta and in the oocyte may be a key driver in the recent evolution of non-canonical and canonical imprinting in extra-embryonic tissues.

Non-canonical imprinting is mediated by inheritance of H3K27me3 from the oocyte and was suggested to maintain a few non-canonical imprints into the extra-embryonic development [21]. In our study, we identified all four previously reported non-canonical imprinted genes [21], in addition to several novel domains. Furthermore, we demonstrated conclusively that non-canonical imprinted genes are mono-allelically expressed independent of inherited maternal DNA methylation. However, we find that maternal enrichment for H3K27me3 does not persist beyond pre-implantation development at non-canonically imprinted loci but rather is replaced by maternal DNA methylation in post-implantation ExE. Conversely, non-canonical imprinted ERVK LTRs become bi-allelically silenced in embryonic lineages by the acquisition of bi-allelic DNA methylation. The mechanisms underlying the transition in repressive epigenetic states on the maternal allele are unclear, and why the allelic specificity would persist in ExE, but not in the epiblast, remains to be explored.

Despite the lack of allelic H3K27me3 at non-canonical imprinted loci in post-implantation ExE, we find a role for imprinted H3K27me3 at other genomic regions. We identified four silenced imprinted genes (*Plagl1*, *Slc22a3*, *Pde10a*, and *Magel2*) where the active allele was demarked by bivalent chromatin in E6.5 ExE, which subsequently resolved to imprinted gene expression in E12.5 placentae. Thus, bivalent chromatin may enable the temporal regulation of imprinted gene expression in extra-embryonic development, similar to that which has been observed in embryonic lineages [38, 39]. We also find large allelic H3K27me3 domains at the *Igf2r/Airn* and *Kcnq1/Kcnq1ot1* loci in extra-embryonic tissues in vivo and identify a number of novel imprinted genes distal of the *Igf2r/Airn* cluster. Furthermore, we show that the maternal gDMRs at these loci are required to prevent bi-allelic acquisition of H3K27me3. These findings support the observations from trophoblast stem cells in vitro that have shown lncRNAs regulated by the *Igf2r/Airn* and *Kcnq1/Kcnq1ot1* maternal gDMRs mediate the recruitment of PRC2 and spreading of H3K27me3 in cis, in an X chromosome inactivation-like mechanism of silencing [40–42].

## Conclusions

Our study of imprinted genes in in vivo post-implantation extra-embryonic development has provided novel insights into non-canonical imprinted gene regulation, which are otherwise masked in bulk genomic data from inbred strains and are difficult to assess in human populations due to the sparsity of genetic polymorphisms. We reveal that the majority of non-canonical imprints are localized to solo ERVK LTR repeats, which act as imprinted transcription initiation sites for non-coding RNAs and chimeric mRNAs in extra-embryonic tissues. Importantly, we find that the regulation of non-canonical imprinted regions transition from inherited maternal H3K27me3 to secondary imprinted DMRs specifically in extra-embryonic lineages. These findings highlight the unique mechanisms regulating imprinted gene expression in the placenta and the potential importance of the still unexplored role of these non-canonical imprints in regulating placentation and fetal growth.

## Methods

### Sample collection

Reciprocal natural timed matings were set up between C57BL6/Babr and CAST/EiJ animals (denoted as B6/CAST and CAST/B6), and embryos were collected on embryonic days 6.5 (E6.5) and 7.5 (E7.5). Natural timed matings were set up between *Dnmt3a* floxed/floxed, *Dnmt3b* floxed/floxed, Zp3+ve B6/129 females (resulting in ablation of DNA methylation in the oocyte) [9] and CAST males (denoted as matDKO/CAST). The epiblast (Epi) and extra-embryonic ectoderm (ExE) for each embryo were manually separated. E6.5 epiblast (*N* = 4) and ExE (*N* = 8) samples were pooled (an estimated ~ 2500 cells), washed in PBS, and then flash-frozen in 10 μL of nuclear lysis buffer (Sigma-Aldrich). Single E7.5 epiblast and ExE samples were individually frozen in 10 μL of buffer RLT Plus (Qiagen).

### In vivo CRISPR targeting

C57BL6/J females were superovulated and crossed with CAST/EiJ males. Zygotes were recovered the next day and electroporated with two sgRNAs for the *Gab1* RLTR15 ERVK element (200 ng/μL each) (Additional file 1: Figure S7) and CAS9 protein (500 ng/μL). Embryos were then implanted into NMRI pseudo-pregnant females. The embryos were dissected on E12.5, and the following tissues were collected: (1) visceral yolk sac/amnion, (2) placental disc with as much decidua removed as possible, (3) embryo, and (4) tail clip for genotyping. Tissue samples were washed in cold PBS and flash-frozen in 50 μL of RLT+ buffer (Qiagen). Tissues were collected from a total of 42 E12.5 embryos from 6 females across 2 independent experiments.

DNA from tail clippings were genotyped using MyTaq master mix (Bioline) with primers: F - AGCCCAATCTCACAACAGTT, R - CGGACCAGGTGAACATGTTG. Bands corresponding to the wild-type (847 bp) and knockout (320 bp) alleles were gel extracted and sent for Sanger sequencing to identify the targeted allele (Additional file 1: Figure S7). One effectively targeted sample (F4E5) and three wild-type controls (F4E1, F4E3, and F5E6) were selected for RNA sequencing.

### mRNA sequencing library preparation

RNA was extracted from E12.5 yolk sac, placenta, and whole embryo from F4E1 (*Gab1* ERVK RLTR15 +/+), F4E3 (*Gab1* ERVK RLTR15 +/+), F4E5 (*Gab1* ERVK RLTR15 +/−), and F5E6 (*Gab1* ERVK RLTR15 +/+) embryos using RNeasy Mini Kit (Qiagen) as per the manufacturer’s instructions. RNA was treated with TURBO DNase (Thermo Fisher Scientific), and quality was assessed with RNA Pico Kit (Agilent) on the Agilent 2100 Bioanalyzer (RIN > 8.7 for all samples). mRNA sequencing libraries were generated using SmartSeq v4 cDNA generation and Nextera XT library preparation, as per manufacturers’ instructions. The quantification of all libraries was done using the High DNA Sensitivity Bioanalyzer 2500 (Agilent) and Illumina library quantification kit (KAPA). Libraries were sequenced using 125 bp paired-end on the Illumina HiSeq 2500 RapidRun, multiplexing 12 samples over 2 lanes.

### Low-input mRNA sequencing library preparation

Stranded mRNA-seq libraries were generated for E7.5 embryos: B6/CAST Epi (*N* = 3) and ExE (*N* = 3), CAST/B6 Epi (*N* = 3) and ExE (*N* = 3), and matDKO/CAST Epi (*N* = 3) and ExE (*N* = 3). Total RNA was extracted using a TRIzol extraction method, as previously described [27]. In brief, samples were homogenized in 400 μL of TRIzol (Invitrogen) and phase-separated by adding 80 μL of chloroform:isoamyl alcohol (Sigma-Aldrich), mixed, and centrifuged at 4 °C for 15 min. The aqueous phase was transferred to a new tube, 1 μL GlycoBlue and 300 μL of ice-cold isopropanol were added and mixed. Samples were incubated for 10 min and then centrifuged for 10 min at 4 °C. The pellet was washed once with 75% ethanol, air-dried, and then resuspended in 5 μL of RNase-free water. Twenty microliters of lysis/binding buffer was immediately added to each RNA sample, and oligo (dT)_25_ capture of mRNA was done using Dynabeads mRNA DIRECT kit (Life Technologies). The protocol was implemented as per manufacturer’s instructions including the additional steps for elimination of rRNA contamination. Maxymum Recovery tubes (Axygen) were used, and volumes were adapted for the low amount of starting material: 5 μL of Dynabeads Oligo (dT)_25_ were used for each sample, mRNA capture was done in a total volume of 50 μL of lysis/binding buffer, washes were done using 100 μL Washing Buffer A or 50 μL of Washing Buffer B, and a final elution volume of 5 μL 10 mM Tris-HCl. The total volume of mRNA was then immediately advanced into the library preparation protocol, using the SMARTer Stranded RNA-seq kit (Clontech), which is optimized for as little as 100 pg of RNA. The protocol was completed as per the manufacturer’s instructions, and 14 amplification cycles were used for all samples. The quantification of all libraries was done using the High DNA Sensitivity Bioanalyzer 2500 (Agilent) and Illumina library quantification kit (KAPA). Libraries were sequenced using 50 bp single-end on the Illumina HiSeq 2500 RapidRun, multiplexing 12 samples per lane. Libraries were evaluated for quality in SeqMonk using RNA-seq QC and duplication plots, resulting in 1 replicate of B6/CAST ExE being excluded due to high duplication.

### Ultra low-input native chromatin immunoprecipitation

Ultra low-input native ChIP-seq was done as previously described [22]. ChIP-seq libraries for H3K4me3, H3K27me3, H3K36me3, and 10% inputs were generated for replicates of pooled E6.5 embryos: B6/CAST Epi (*N* = 2) and ExE (*N* = 2), CAST/B6 Epi (*N* = 2) and ExE (*N* = 2), and matDKO/CAST Epi (*N* = 2) and ExE (*N* = 2). Prior to the immunoprecipitation with antibody-bound beads, each chromatin sample (200 μL) was divided into 5 aliquots: 1 for each antibody (54 μL), 1 10% input (20 μL), and 1 10% input for bisulphite sequencing (20 μL) (Additional file 1: Figure S1). For each immunoprecipitation, 250 ng of anti-H3K4me3 (Diagenode K02921004), 125 ng of anti-H3K27me3 (Millipore 07-449), and 250 ng of anti-H3K36me3 (Diagenode C15410192) were used. Library preparation was done using the MicroPlex Library Preparation kit v2 (Diagenode), as per the manufacturer’s recommendations, and libraries amplified using 15 amplification cycles. Quantification was done using the High DNA Sensitivity Bioanalyzer 2500 (Agilent) and Illumina library quantification kit (KAPA). Samples were multiplexed using 75 bp paired-end sequencing on Illumina NextSeq500.

### Low-input post-bisulphite adaptor tagging from ChIP input samples

Post-bisulphite adaptor tagging (PBAT) was done on 10% input samples, as previously described [22]. A corresponding 10% input was taken from each ChIP sample above, with the addition of replicates, where available: B6/CAST Epi (*N* = 3) and ExE (*N* = 2), CAST/B6 Epi (*N* = 3) and ExE (*N* = 2), and matDKO/CAST Epi (*N* = 3) and ExE (*N* = 3). All input PBAT libraries were amplified using 12 amplification cycles; quantification was done using the High DNA Sensitivity Bioanalyzer 2500 (Agilent) and Illumina library quantification kit (KAPA). Samples were multiplexed using 75 bp paired-end sequencing on Illumina NextSeq500. One replicate of matDKO/CAST epiblast was excluded from the analysis due to < 5% unique mappability.

### Post-bisulphite adaptor tagging for deep sequencing

Post-bisulphite adaptor tagging (PBAT) was done on E7.5 epiblast and ExE samples from single embryos (B6/CAST Epi and ExE (*N* = 2), CAST/B6 Epi and ExE (*N* = 2)), as previously described [43]. Quantification was done using the High DNA Sensitivity Bioanalyzer 2500 (Agilent) and Illumina library quantification kit (KAPA). Samples were multiplexed using 150 bp paired-end sequencing on Illumina NextSeq500, multiplexing four samples per lane.

### Public datasets

The following public datasets were used in this manuscript: stranded total RNA-seq from FvB x CAST reciprocal hybrids for E12.5 placenta, E12.5 visceral endoderm, E12.5 liver, E16.5 brain, E16.5 heart, and E16.5 liver [15]; H3K27me3 ChIP-seq from B6 x PWK hybrid embryos for early and late 2-cell embryos, 8-cell embryos, and E3.5 blastocyst inner cell mass (ICM) [31]; H3K4me3 ChIP-seq from B6 x PWK hybrid embryos for early and late 2-cell embryos, 8-cell embryos, and E3.5 blastocyst inner cell mass (ICM) [44]; RNA-seq and bisulphite-seq from C57BL/6 pre- and post-implantation embryos [30]; bisulphite-seq [28], total RNA-seq [27], and H3K4me3 and H3K27me3 ChIP-seq [22] data from C57BL/6 GV oocytes. All raw data files were obtained from Gene Expression Omnibus or the DNA data bank of Japan and were mapped and evaluated using the following pipelines.

### Allelic mapping of sequencing data

RNA-seq data was subjected to trimming with Trim Galore (v0.4.5) and aligned using HISAT2 (v2.1.0, guided by gene models from Ensembl annotation release 70, options: --dta –sp 1000,1000). ChIP-seq and input material was also trimmed with Trim Galore and aligned using Bowtie2 (v2.3.2, options -X 1200). In addition to adapter and quality trimming, bisulfite sequencing data had the first 9 bp of both read 1 and read 2 removed to reduce biases arising from the 9 N oligo pull-down reaction (Trim Galore options: --clip_r1 9 --clip_r2 0 --paired). The trimmed PBAT data was then aligned using Bismark (v0.19.0, options: --pbat) [45].

Following alignments, all sequencing data was then split allele-specifically using SNPsplit (v0.3.3) [46]. In brief, sequencing reads were mapped to a *Mus musculus* (GRCm38)-derived genome, where SNPs between hybrid strains (C57BL6 and CAST/EiJ, or FvB and CAST/EiJ, or C57BL6 and PWK) had been masked by the ambiguity nucleobase N (N-masked genome). Aligned reads were then sorted into one of three BAM files: C57BL6 (genome 1), CAST/EiJ (genome 2), or unassigned. The females carrying conditional *Dnmt3a*/*Dnmt3b* double knockout (matDKO) were predominantly C57BL6; however, there was approximately 15% of 129 alleles remaining in the strain. Therefore, these data were run through a unique pipeline to allow for a complete mapping of the maternal allele. All datasets generated from matDKO/CAST embryos were first aligned to a B6/CAST N-masked genome, as above, but with the difference that all SNPs which were in common between 129 and CAST (~ 2 million) had been excluded. The data was then split against C57BL6 and CAST/EiJ, as above. FastQ reads of the unassigned fraction of reads were then recovered from the original FastQ files, and in the second step, these reads were then aligned to a 129S1/CAST genome (generated with SNPsplit_genome_preparation). Alignments were then SNPplit between 129 and CAST/EiJ, and the 129-specific reads were then combined with the C57BL6-specific reads (from step 1) to comprise a complete maternal allelic set. Raw sequencing reads and allelically mapped BAM files have been deposited into the Gene Expression Omnibus database (GSE124216).

### ChIP-seq peak calling

Peak calling was done for B6/CAST and CAST/B6 epiblast and ExE H3K4me3, H3K27me3, and H3K36me3 using chromstaR, a multivariate peak-calling approach based on a multivariate hidden Markov model, using the default parameters [47].

### Allelic histone enrichment and gene expression analyses

Read counts for maternal and paternal H3K4me3 or H3K27me3 were quantitated over B6/CAST and CAST/B6 H3K4me3 peaks for either epiblast or ExE. H3K4me3 or H3K27me3 peaks were combined and de-duplicated between the B6/CAST and CAST/B6 epiblast or ExE, to generate a complete list of peaks from both hybrid crosses for each tissue. Peaks were then filtered for those with a minimum read count of 20 in at least 1 allelically mapped biological replicate. Peaks with allelically biased H3K4me3 or H3K27me3 were then identified using EdgeR statistic (*p* < 0.05, corrected for multiple comparisons). Significant peaks were then classified into strain-specific allelic H3K4me3 or H3K27me3 if their allelic enrichment switched in the reciprocal cross. Significant peaks were identified as imprinted if the allelic enrichment for a parental allele was consistent between reciprocal crosses.

Read counts for maternal and paternal H3K36me3 or RNA-seq were quantitated over autosomal genes. Genes were then filtered for those with a minimum read count of 20 in at least 1 allelically mapped biological replicate of B6/CAST and CAST/B6 H3K36me3 or a minimum read count of 5 in at least 1 allelically mapped biological replicate of B6/CAST and CAST/B6 (or F/CAST and CAST/F for E12.5 placenta) RNA-seq. Genes with allelically biased expression were then identified using EdgeR statistic (*p* < 0.05, corrected for multiple comparisons). Significant genes were filtered for those that were associated with an imprinted H3K4me3 peak.

### ChIP-seq quantitation

For the quantitative display of allelic ChIP-seq data for a single histone mark and allelic ratios, read counts per running window or peak (where applicable) were averaged between biological replicates. Read counts were normalized to library size, excluding X, Y, and mitochondrial chromosomes, using size-factor normalization in SeqMonk. However, for the comparison of allelic enrichment for H3K4me3 and H3K27me3 across pre-implantation development (Additional file 1: Figure S11B and C), raw read counts were used. For this comparison, correcting for library size is not appropriate, as there is a known discrepant abundance of H3K27me3 between the maternal and paternal alleles [31]. When ChIP-seq data of multiple histone marks are displayed together in a screenshot, enrichment normalized reads per kilobase per million (RPKM) was used. Enrichment normalization performs an initial additive translation of the data based on a low data percentile (40th percentile) representing non-zero but unambiguously unenriched points, followed by a multiplicative expansion of the data to a second high percentile (99th percentile) representing the most highly enriched regions. Enrichment is therefore scaled between these two points, but following the relative enrichment levels seen in the untransformed data.

### Transcript analysis of non-canonical H3K4me3 associated ERVK LTRs

Coordinates for repetitive elements for the mouse GRCm38 genome build were generated using RepeatMasker. Active ERVK LTRs were identified as those that fell within an H3K4me3 peak in E6.5 ExE, with ≥ 5 reads on the same strand at the LTR repeat in at least 2 replicates of RNA-seq data from E7.5 ExE, E12.5 visceral endoderm [15], and/or E12.5 placenta [15] with at least 1 intron-spanning read indicative of spliced RNA. These were then filtered for those that were sites of transcription initiation with no apparent upstream intron-spanning reads (*N* = 40), of which 8 were within non-canonically imprinted paternal H3K4me3 peaks (Additional file 2: Table S5) and 32 were classified as extra-embryonic active ERVK LTRs (Additional file 2: Table S6). The presence and directionality of reads spanning annotated introns, potential introns between annotated and upstream novel exons, and between upstream novel exons were analyzed to determine those ERVK LTRs that were initiating mRNA chimeras or non-coding RNAs (Additional file 2: Tables S5 and S6).

### Sequence motif analysis of extra-embryonic ERVK LTR promoters

DREME (version 5.0.5) was used to identify transcription factor motifs among ERVK LTRs that are transcriptionally active in extra-embryonic tissues (*N* = 40) compared to a background set with comparable length and class. AME (version 5.0.5) was used to evaluate enrichment for transcription factor binding sites for CDX2, EOMES, and ELF5.

## Supplementary information

**Additional file 1.** Supplementary figure and table legends, **Figures. S1-S11.**

**Additional file 2.** Supplementary **Tables S1-S6.**

**Additional file 3.** Review history.

## Data Availability

All data generated for this study is available on Gene Expression Omnibus (GSE124216) [48]. Publically available datasets were sourced from Gene Expression Omnibus (GSE75957 [15], GSE76687 [31], GSE71434 [44], GSE98149 [30], GSE70116 [27], and GSE93941 [22]) and the DNA Data Bank of Japan (DRA000570) [28].
