# Supplementary material for: Endogenous retroviral insertions drive non-canonical imprinting in extra-embryonic tissues
Source: Genome Biol. 2019 Oct 29;20:225. doi: 10.1186/s13059-019-1833-x (PMC6819472; doi:10.1186/s13059-019-1833-x)

Supplementary Figure Legends

Fig S1. A) Scatterplots between biological replicates for H3K4me3, H3K27me3 and H3K36me3 ChIP-seq in B6/CAST, matDKO/CAST and CAST/B6 E6.5 epiblast and ExE. RPKM quantitation of genome-wide 5kb running windows was used. Windows on X, Y and mitochondrial chromosomes were excluded, in addition to genomic regions with mapping artefacts in input control samples (RPKM>4). **B)** A screenshot of H3K4me3, H3K36me3, and H3K27me3 ChIP-seq replicates in B6/CAST E6.5 epiblast and ExE. Enrichment normalised RPKM was quantitated for 5kb running windows. **C)** Hierarchical clustering of H3K4me3, H3K36me3, H3K27me3 and input control ChIP-seq replicates for B6/CAST, CAST/B6 and matDKO/CAST E6.5 epiblast and ExE. Quantitation is as described in A.

Fig S2. A) Principle component analysis of genome-wide DNA methylation profiles of B6/CAST, CAST/B6 and matDKO/CAST E6.5 epiblast and ExE replicates. DNA methylation was quantitated over 500-CpG running windows on autosomes, with at least 20 informative CpGs per window. **B)** DNA methylation values are shown for maternal imprinted gDMRs (N=21) of grouped replicates for B6/CAST, CAST/B6 and matDKO/CAST E6.5 epiblast and ExE. DNA methylation was quantitated over DMR regions, with at least 10 informative CpGs. **C)** Screenshot of DNA methylation profiles of B6/CAST, CAST/B6 and matDKO/CAST E6.5 epiblast and ExE replicates. DNA methylation was quantitated as in A.

Fig S3. A) Principle component analysis of gene expression in B6/CAST, CAST/B6 and matDKO/CAST E7.5 epiblast and ExE replicates. Gene expression was quantitated as log_2RPKM of autosomal genes. **B)** Correlation matrix between RNA-seq replicates for B6/CAST, CAST/B6 and matDKO/CAST E7.5 epiblast and ExE. Gene expression was quantitated as in A. **C)** Scatterplots between E7.5 epiblast and ExE in B6/CAST, CAST/B6 and matDKO/CAST. Gene expression was quantitated as in A. Labelled genes are known to be preferentially expressed in epiblast (*Pou5f1*) and ExE (*Elf5, Esrrb, Tfap2c*). **D)** Boxplots depict the enrichment of genic H3K36me3, promoter H3K4me3 and promoter H3K27me3

(E6.5) for each decile of gene expression (E7.5) in B6/CAST ExE (top) and epiblast (bottom). Histone enrichment was quantitated as log_2RPKM, correcting for probe length. Genic H3K36me3 is positively correlated ($p<2.2E-16$), promoter H3K4me3 is positively correlated ($p<2.2E-16$), and promoter H3K27me3 is anti-correlated ($p<2.2E-16$) with gene expression in both ExE and epiblast.

Fig S4. A) Scatter plots of allelic H3K4me3 enrichment at informative autosomal H3K4me3 peaks (N=15,975) in B6/CAST epiblast (left) and CAST/B6 epiblast (right). Peaks with allelically-biased H3K4me3 were identified using EdgeR statistic ($p<0.05$, corrected for multiple comparisons). Significant peaks were then classified into strain-specific allelic H3K4me3 if their allelic enrichment switched in the reciprocal cross, denoted as B6-specific (green) and CAST-specific (turquoise). Significant peaks were identified as imprinted if the allelic enrichment was consistent between reciprocal crosses, denoted as paternal (blue) or maternal (red). Enrichment is quantitated as read count normalised to library size, correcting for probe length. **B)** Heatmap showing allelic bias (log_2(pat/mat)) for H3K4me3 in E6.5 epiblast (Epi) at imprinted H3K4me3 peaks identified in A. Allelic bias for H3K36me3 in E6.5 Epi and gene expression in E7.5 Epi are shown for associated genes using reciprocal hybrids. Reciprocal hybrids are denoted as B/C (B6/CAST) and C/B (CAST/B6). White boxes indicate where there was insufficient data (ChIP-seq: <20 SNP-spanning reads in all replicates, RNA-seq: <5 SNP-spanning reads in all replicates). ChIP-seq data was quantitated as read count normalised to library size, RNA-seq data was quantitated as read count over exons. H3K4me3 peaks were excluded if there was no gene within 10kb or the associated gene was uninformative in all datasets. H3K4me3 peaks associated with more than one gene are duplicated in the H3K4me3 column. Novel genes are marked with an asterisk. **C)** Screenshot of allelic enrichment for H3K4me3 and H3K36me3 in E6.5 epiblast and gene expression in E7.5 epiblast for B6/CAST and CAST/B6 at the known imprinted locus *Peg3*. ChIP-seq data is quantitated using enrichment normalised RPKM (scales in square brackets), paternal (blue) and maternal (red) enrichment are shown on mirrored axes. Gene expression is quantitated using RPKM for 1kb running windows with a 100bp step.

Fig S5. A) Scatter plot of allelic H3K4me3 enrichment at informative autosomal H3K4me3 peaks (N=15,975) in matDKO/CAST E6.5 ExE. Peaks with allelically-biased H3K4me3 were identified using EdgeR statistic ($p<0.05$, corrected for multiple comparisons). Significant peaks were then classified into strain-specific allelic H3K4me3 if their allelic enrichment switched in the reciprocal cross, denoted as B6-specific (green) and CAST-specific (turquoise). Significant peaks were identified as imprinted if the allelic enrichment was consistent between reciprocal crosses, denoted as paternally-regulated (blue) or non-canonical (red). Enrichment is quantitated as read count normalised to library size, correcting for probe length. **B)** The percentage of imprinted H3K4me3 peaks overlapping ERV LTRs (non-canonical [n=14] and canonical [N=20]) overlapping each class of ERV repeats. Each pair-wise comparison was done using Chi-Square statistic, with a significance threshold adjusted for multiple comparisons using Bonferroni correction. **C)** Screenshots of directional RNA-seq reads for CAST/F E12.5 embryonic (liver, brain, heart) and extra-embryonic (placenta, VE – visceral endoderm) tissues. Boxes highlight each of the non-canonically imprinted active ERVK LTRs (N=8). Reads and genes are shown in red for + strand and blue for – strand.

Fig S6. Beanplot shows the distribution of lengths of all mappable ERVK LTRs (N=334,325) with ERVK LTRs active in extra-embryonic tissues, including non-canonically imprinted ERVKs, shown in blue dots (N=40).

Fig S7. A) Screenshot of allelic directional RNA-seq intron-spanning reads in E12.5 placenta from FvB (F)/CAST and CAST/F reciprocal hybrids for the *Gab1* locus. Raw intron-spanning reads are shown in blue for the – strand and red for the + strand. Box depicts the non-canonical paternal H3K4me3 peak containing ERVK RLTR15, which acts as an alternative promoter for the *Gab1* gene. **B)** Schematic diagram showing the location of repetitive regions, CAST and JF1 SNPs for experimental design, and the location of CRISPR gRNAs used to excise RLTR15 within intron 1 of the *Gab1* gene.

The two SNPs used for genotyping the deleted allele are labelled (rs4618180 and rs235146078). **C)** Image of 2% agarose gel with amplified WT (847bp) and KO (320bp) alleles for tissues (yolk sac, placenta and whole embryo) from CRISPR-targeted E12.5 embryos used for RNA-sequencing (F4E1, F4E3, F4E5, F5E6). **D)** Peak trace chromatograms showing B6/CAST SNPs rs4618180 and rs235146078 for C57BL/6 control and the F4E5 KO allele (320bp), demonstrating the deletion of RLTR15 is on the CAST (paternal) allele. **E)** Hierarchical clustering of RNA-seq libraries from E12.5 B6/CAST tissues (yolk sac, placenta and whole embryo) from F4E1 (wildtype – WT), F4E3 (WT), F4E5 (mosaic *Gab1* RLTR15 +/-), F5E6 (WT) embryos. Autosomal genes were quantitated using FPKM. **F)** Barplot shows the allelic gene expression (allelic ratio = mat/(mat+pat)) for the control gene *Sfmbt2*, a non-canonically imprinted gene, in B6/CAST E12.5 yolk sac, placenta and whole embryos. F4E5 carried CRISPR-targeted deletion of non-canonically imprinted *Gab1* RLTR15 on the paternal allele and was compared to wildtype (WT) controls (N=3). Two-tailed single sample t-test was used to compare the F4E5 value to the WT mean (* = $p<0.05$). Error bars show standard deviation.

Fig S8. A) Screenshot of allelic gene expression across the *Slc38a4* locus in E7.5 B6/CAST, CAST/B6 and matDKO/CAST epiblast and ExE. Expression (RPKM) is displayed for running 500bp windows with 50bp step. The canonical maternal gDMR promoter and non-canonical imprinted paternal ERVK LTR element are noted in the labelled boxes. **B)** Screenshot of allelic H3K4me3 in B6/CAST, CAST/B6 and matDKO/CAST ExE and epiblast across the *Slc38a4* locus. H3K4me3 was quantitated as read count normalised to library size, using 500bp running windows with 50bp step (scales in square brackets). Paternal (blue) and maternal (red) enrichment are shown on mirrored axes. **C)** Screenshot of gene expression, H3K4me3, H3K27me3, and DNA methylation in C57BL/6 GV oocytes across the *Slc38a4* locus. All data is shown using 1kb running windows with 100bp step, ChIP-seq data is quantitated as enrichment normalised RPKM (scales in square brackets). DNA methylation is quantitated using a minimum coverage of at least one CpG per 1kb window. The box labelled oocyte promoter(s) depicts

several H3K4me3-marked MaLR insertions upstream of the *Slc38a4* gDMR promoter, which derive oocyte-specific transcription across the gDMR promoter.

Fig S9. A) Scatter plot showing allelic H3K27me3 enrichment of 5kb running windows (tiles), excluding X and Y chromosomes, in CAST/B6 E6.5 ExE (left), B6/CAST E6.5 ExE (middle) and B6/CAST E6.5 epiblast (right). Quantitation is average read count per window, normalised to library size, using two biological replicates for each tissue. 5kb running windows that overlapped paternal and maternal imprinted H3K27me3 peaks, identified as described for H3K4me3 peaks, are highlighted in blue and red, respectively. **B)** Screenshot of allelic H3K27me3 in B6/CAST, CAST/B6 and matDKO/CAST E6.5 ExE (top) and epiblast (bottom), with the paternal allelic enrichment in blue and maternal in red on mirrored axes. H3K27me3 was quantitated using autosomal 5kb running windows, as read count normalised to library size (scales in square brackets). The maternal gDMR within the locus is denoted by a black bar. Constitutive imprinted genes are labelled in black, and known, putative and novel placental-specific imprinted genes in green. Imprinted genes with paternal allele-specific expression are coloured blue and maternal allele-specific expression are red. Annotation tracks for imprinted H3K27me3 and H3K4me3 peaks are shown by purple and orange bars, respectively, for ExE (top panel) and epiblast (bottom panel).

Fig S10. Screenshot of allelic H3K27me3 in B6/CAST, CAST/B6 and matDKO/CAST E6.5 ExE (top) and epiblast (bottom) at the *Kcnq1/Kcnq1ot1* locus, with the paternal allelic enrichment in blue and maternal in red on mirrored axes. H3K27me3 was quantitated using autosomal 5kb running windows, as read count normalised to library size (scales in square brackets). The maternal (red) and paternal (blue) gDMR within the locus are denoted by black bars. Known imprinted genes are labelled in black, and known, putative and novel placental-specific imprinted genes in green. Imprinted genes with paternal allele-specific expression are coloured blue and maternal allele-

specific expression are red. Annotation tracks for imprinted H3K27me3 and H3K4me3 peaks are shown by purple and orange bars, respectively, for ExE (top panel) and epiblast (bottom panel).

Fig S11. A) Heatmap showing DNA methylation for the maternal and paternal allele in B6/CAST and CAST/B6 E7.5 epiblast at non-canonically imprinted active ERVK LTRs±500bp (N=8). White boxes are where these was insufficient data in at least one of the replicates. **B)** Scatterplots of allelic H3K27me3 at H3K4me3 peaks (defined in E6.5 ExE) across pre- and post-implantation development (late 2-cell embryos, E3.5 inner cell mass (ICM), and E6.5 epiblast and ExE). Non-canonical imprinted paternal H3K4me3 peaks are highlighted in blue. Allelic H3K27me3 enrichment is quantitated as read count corrected for peak length. **C)** Scatterplots of allelic H3K4me3 at H3K4me3 peaks (defined in E6.5 ExE) across pre- and post-implantation development (late 2-cell embryos, E3.5 inner cell mass (ICM), and E6.5 epiblast and ExE). Non-canonical imprinted paternal H3K4me3 peaks are highlighted in blue. Allelic H3K4me3 enrichment is quantitated as in B.

Supplementary Table Legends

Table S1. Details of all datasets generated for this study, including ultra-low input ChIP-seq, RNA-seq and bisulphite-seq.

Table S2. H3K4me3 peaks that show mono-allelic imprinted enrichment in ExE, their associated gene(s), and whether the associated genes are known, putative or novel, based on Table S4. Each gene was evaluated for significant allelic bias using EdgeR statistical approach ($p<0.05$ corrected for multiple comparisons) in reciprocal hybrids for E6.5 ExE H3K36me3, E7.5 ExE gene expression, and E12.5 placenta gene expression – classified as yes, no or ND (ND = no data, where there was insufficient SNP-spanning reads).

Table S3. H3K4me3 peaks that show mono-allelic imprinted enrichment in epiblast, their associated gene(s), and whether the associated genes are known, putative or novel, based on Table S4. Each gene was evaluated for significant allelic bias using EdgeR statistical approach ($p<0.05$ corrected for multiple comparisons) in reciprocal hybrids for E6.5 epiblast H3K36me3 and E7.5 epiblast gene expression – classified as yes, no or ND (ND = no data, where there was insufficient SNP-spanning reads).

Table S4. List of known imprinted genes based on those listed in Mousebook (www.mousebook.org/). Genes were listed as putatively imprinted if they were identified to be imprinted in placental trophoblast (and/or constitutively) by the Otago catalogue of parental origin effects (http://igc.otago.ac.nz/) and/or references from the literature.

Table S5. ERVK LTR localisation and transcriptional activity at non-canonical paternal H3K4me3 peaks. ERVK LTR transcription is confirmed if the LTR element had a read count ≥5 in at least two replicates of reciprocal hybrid E7.5 ExE (N=5), E12.5 placenta (N=4) and/or E12.5 visceral endoderm (N=4). ERVK LTR location, allelic bias in expression, and promoter activity for a non-coding RNA or chimeric mRNA, defined as intron-spanning reads extending to an exon of an annotated nearby gene(s) is specified.

Table S6. Non-imprinted ERVK LTRs with promoter activity (site of transcription initiation) in extra-embryonic tissues, defined as a read count ≥5 in at least two replicates of reciprocal hybrid E7.5 ExE (N=5), E12.5 placenta (N=4) and/or E12.5 visceral endoderm (N=4). These were then subsequently filtered for those that were site of transcription initiation, determined by intron-spanning reads. ERVK LTR location and promoter activity for a non-coding RNA or chimeric mRNA, defined as intron-spanning reads extending to an exon of an annotated nearby gene(s), is specified.

Supplementary Figure 1. E6.5 embryo ChIP-seq QC

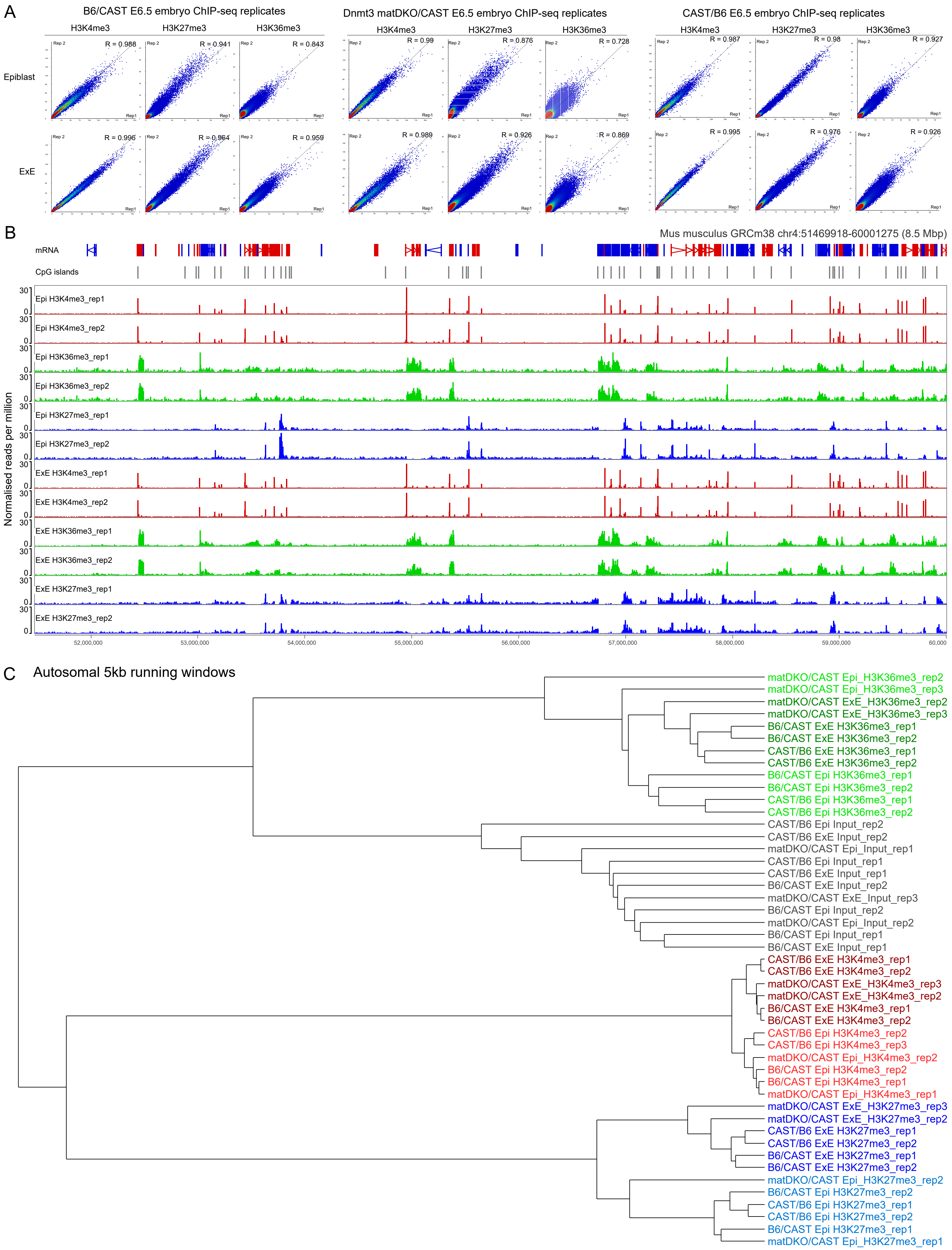

Supplementary Figure 2

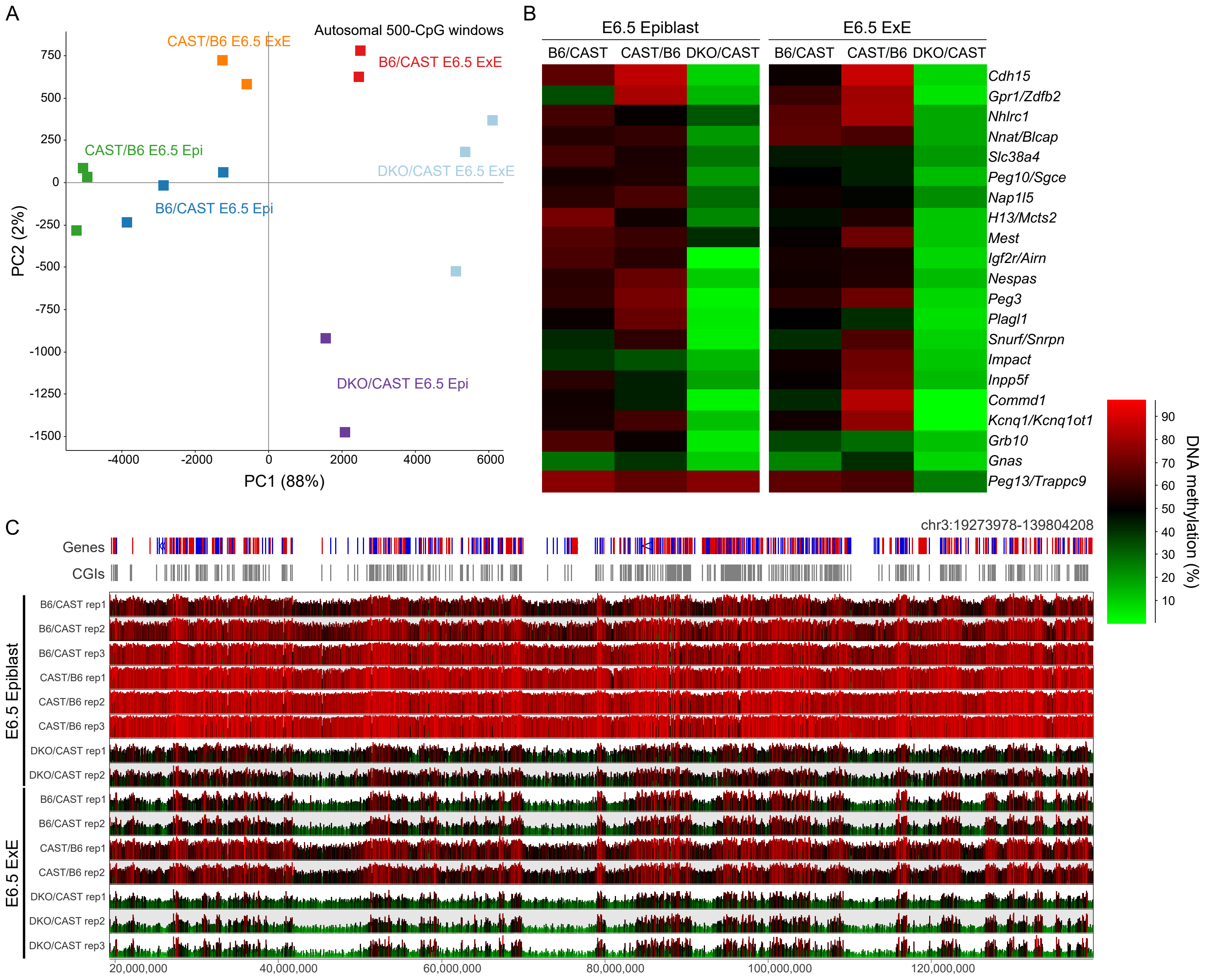

Supplementary Figure 3

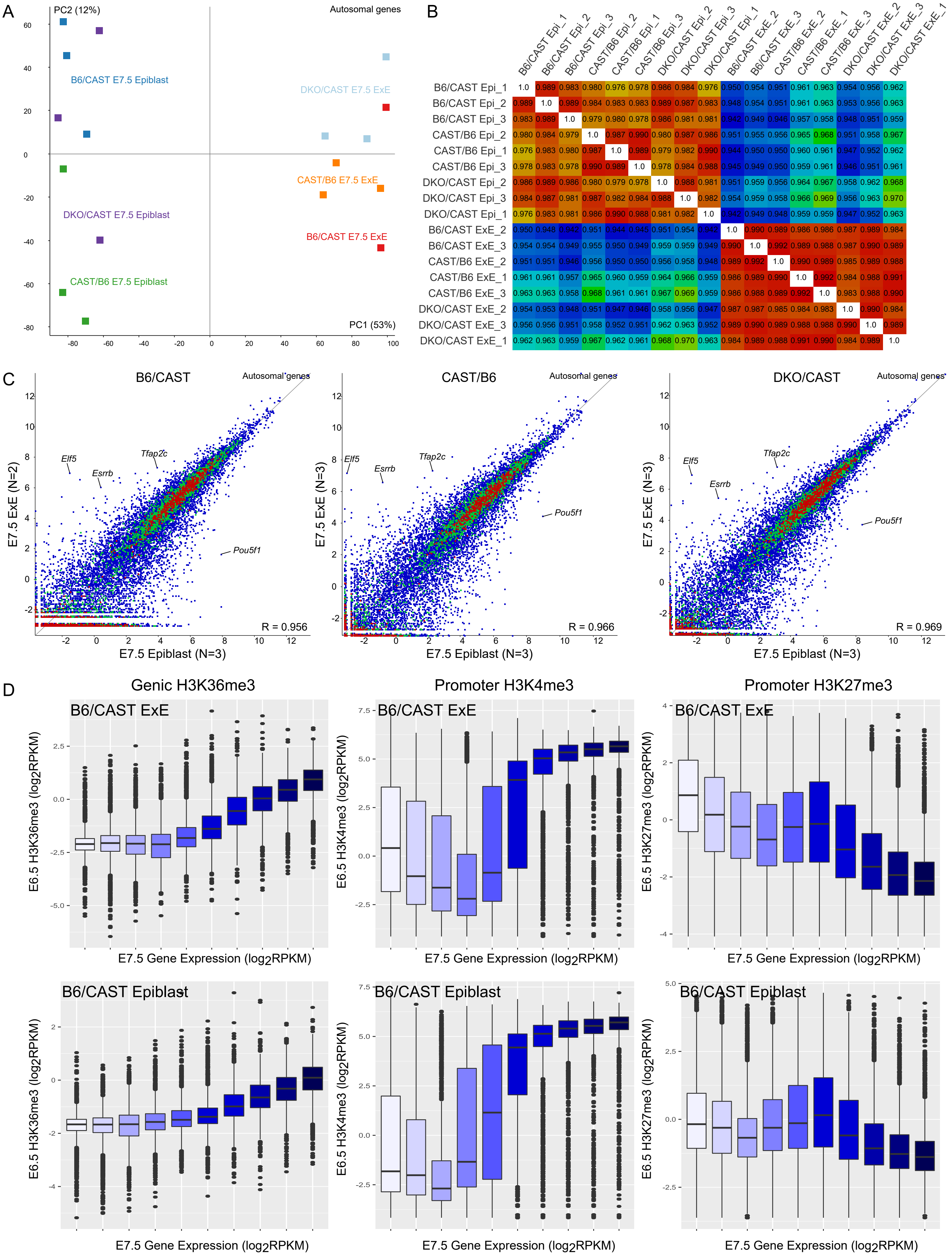

	B6/CAST Epi_1	B6/CAST Epi_2	B6/CAST Epi_3	CAST/B6 Epi_2	CAST/B6 Epi_1	CAST/B6 Epi_3	DKO/CAST Epi_2	DKO/CAST Epi_3	DKO/CAST Epi_1	B6/CAST ExE_2	B6/CAST ExE_3	CAST/B6 ExE_2	CAST/B6 ExE_1	CAST/B6 ExE_3	DKO/CAST ExE_2	DKO/CAST ExE_3	DKO/CAST ExE_1
B6/CAST Epi_1	1.0	0.989	0.983	0.980	0.976	0.978	0.986	0.984	0.976	0.950	0.954	0.951	0.961	0.963	0.954	0.956	0.962
B6/CAST Epi_2	0.989	1.0	0.989	0.984	0.983	0.983	0.989	0.987	0.983	0.948	0.954	0.951	0.961	0.963	0.953	0.956	0.963
B6/CAST Epi_3	0.983	0.989	1.0	0.979	0.980	0.978	0.986	0.981	0.981	0.942	0.949	0.946	0.957	0.958	0.948	0.951	0.959
CAST/B6 Epi_2	0.980	0.984	0.979	1.0	0.987	0.990	0.980	0.987	0.986	0.951	0.955	0.956	0.965	0.968	0.951	0.958	0.967
CAST/B6 Epi_1	0.976	0.983	0.980	0.987	1.0	0.989	0.979	0.982	0.990	0.944	0.950	0.950	0.960	0.961	0.947	0.952	0.962
CAST/B6 Epi_3	0.978	0.983	0.978	0.990	0.989	1.0	0.978	0.984	0.988	0.945	0.949	0.950	0.959	0.961	0.946	0.951	0.961
DKO/CAST Epi_2	0.986	0.989	0.986	0.980	0.979	0.978	1.0	0.988	0.981	0.951	0.959	0.956	0.964	0.967	0.958	0.962	0.968
DKO/CAST Epi_3	0.984	0.987	0.981	0.987	0.982	0.984	0.988	1.0	0.982	0.954	0.959	0.958	0.966	0.969	0.956	0.963	0.970
DKO/CAST Epi_1	0.976	0.983	0.981	0.986	0.990	0.988	0.981	0.982	1.0	0.942	0.949	0.948	0.959	0.959	0.947	0.952	0.963
B6/CAST ExE_2	0.950	0.948	0.942	0.951	0.944	0.945	0.951	0.954	0.942	1.0	0.990	0.989	0.986	0.986	0.987	0.989	0.984
B6/CAST ExE_3	0.954	0.954	0.949	0.955	0.950	0.949	0.959	0.959	0.949	0.990	1.0	0.992	0.989	0.988	0.987	0.990	0.989
CAST/B6 ExE_2	0.951	0.951	0.946	0.956	0.950	0.950	0.956	0.958	0.948	0.989	0.992	1.0	0.990	0.989	0.985	0.989	0.988
CAST/B6 ExE_1	0.961	0.961	0.957	0.965	0.960	0.959	0.964	0.966	0.959	0.986	0.989	0.990	1.0	0.992	0.984	0.988	0.991
CAST/B6 ExE_3	0.963	0.963	0.958	0.968	0.961	0.961	0.967	0.969	0.959	0.986	0.988	0.989	0.992	1.0	0.983	0.988	0.990
DKO/CAST ExE_2	0.954	0.953	0.948	0.951	0.947	0.946	0.958	0.956	0.947	0.987	0.987	0.985	0.984	0.983	1.0	0.990	0.984
DKO/CAST ExE_3	0.956	0.956	0.951	0.958	0.952	0.951	0.962	0.963	0.952	0.989	0.990	0.989	0.988	0.988	0.990	1.0	0.989
DKO/CAST ExE_1	0.962	0.963	0.959	0.967	0.962	0.961	0.968	0.970	0.963	0.984	0.989	0.988	0.991	0.990	0.984	0.989	1.0

Supplementary Figure 4

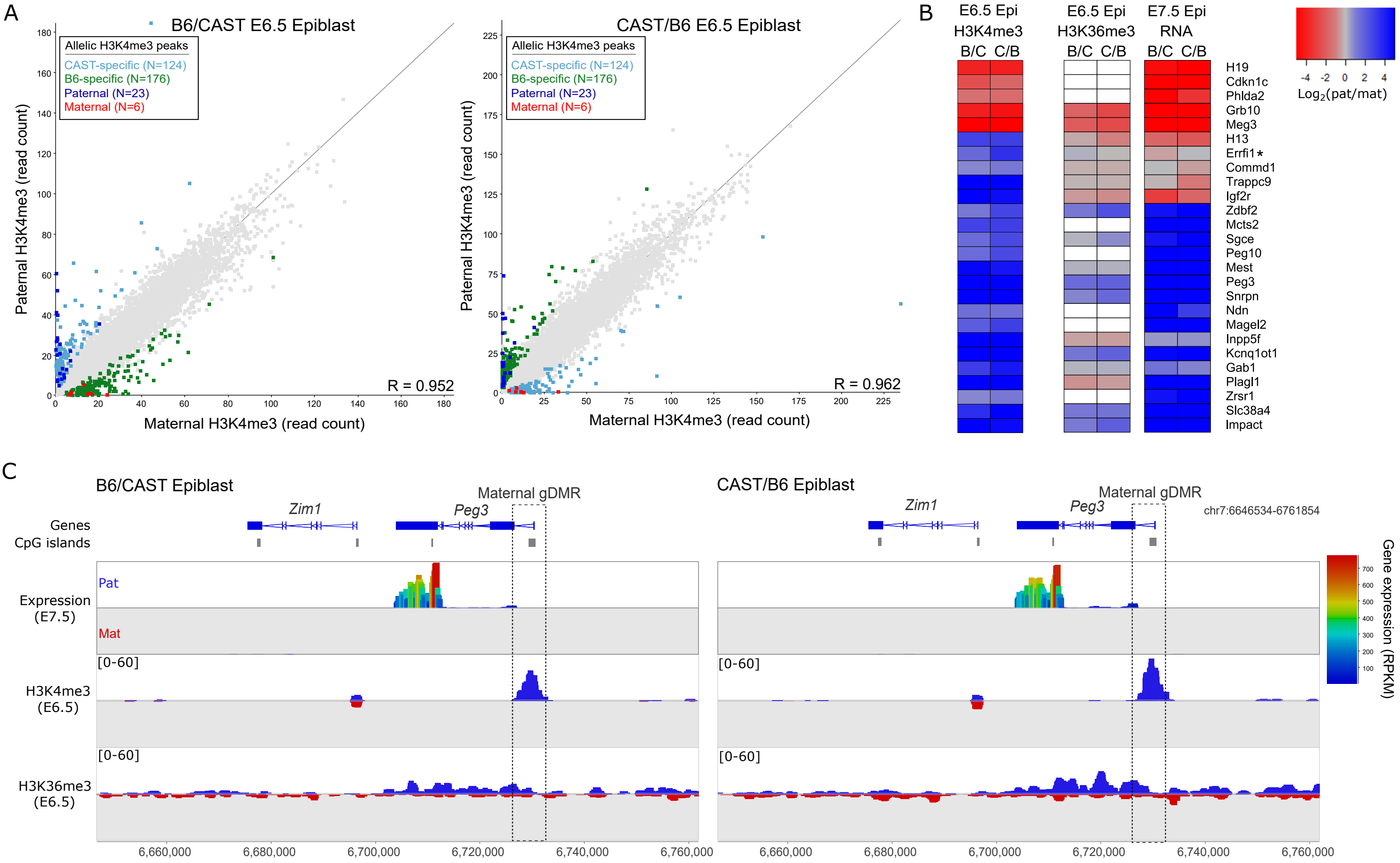

Supplementary Figure 5

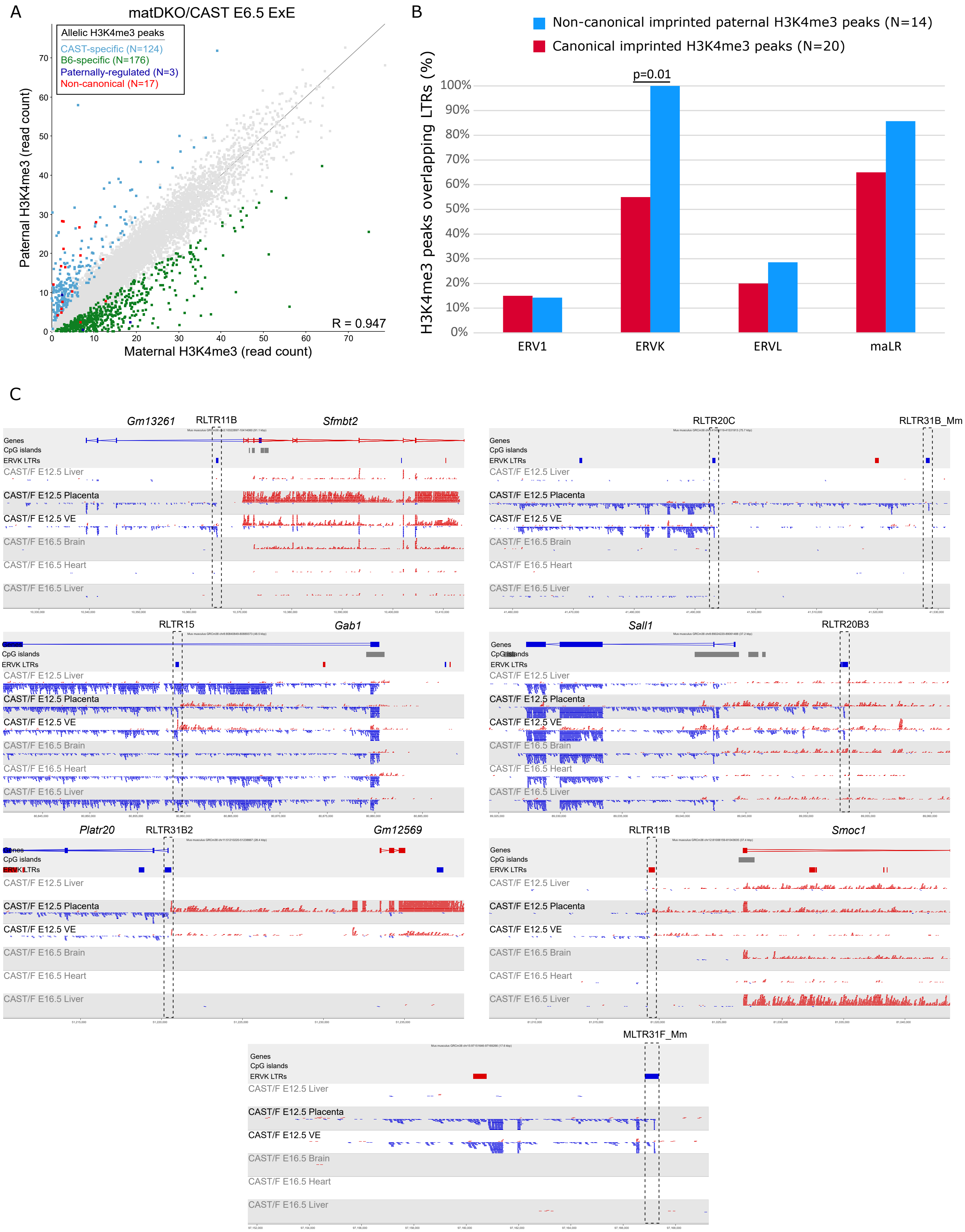

Supplementary Figure 6

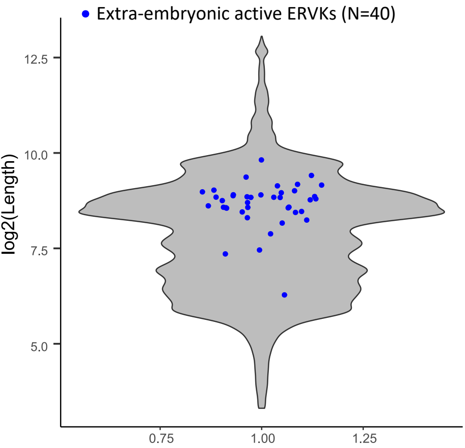

Supplementary Figure 7

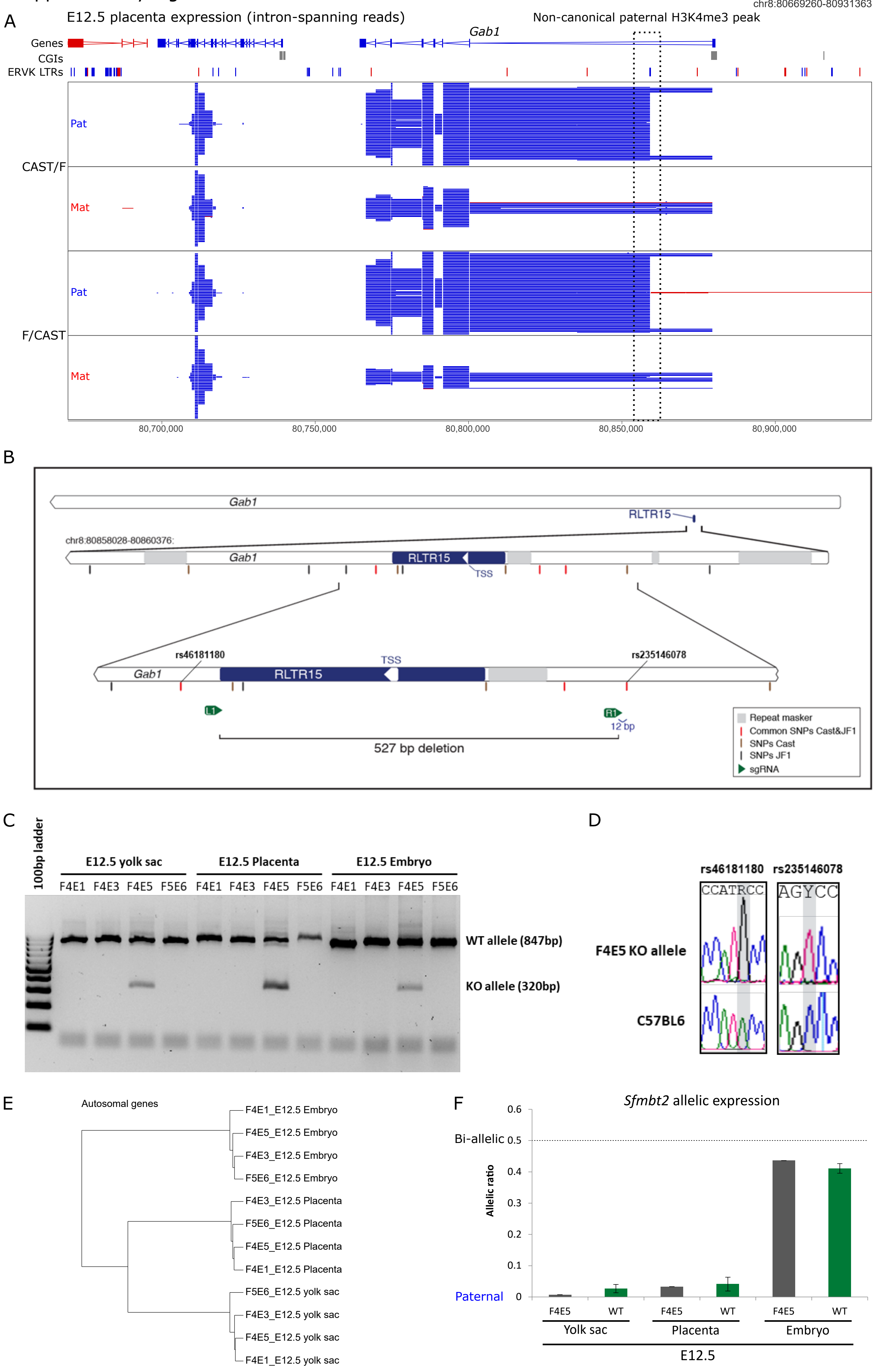

Supplementary Figure 8

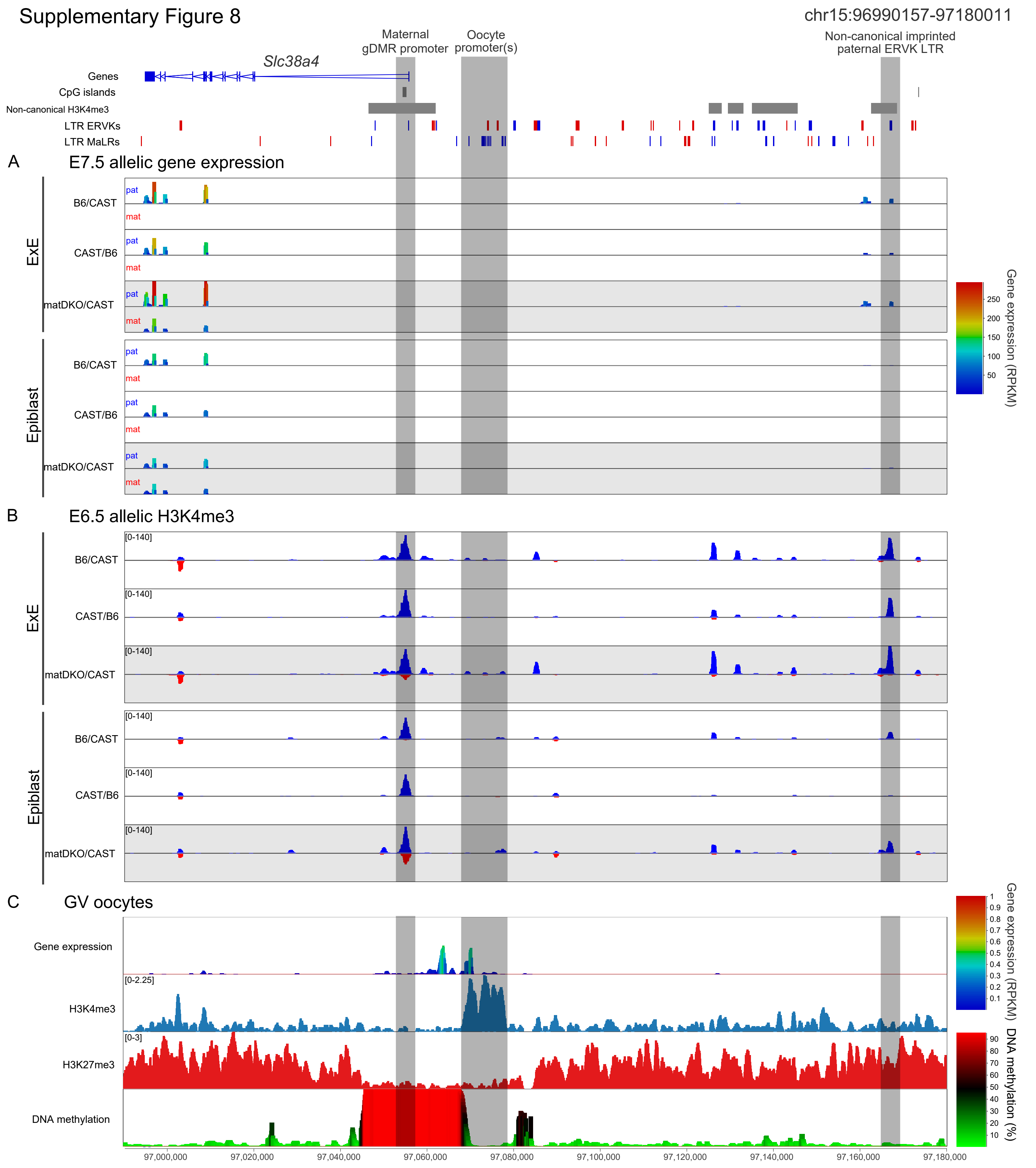

Supplementary Figure 9

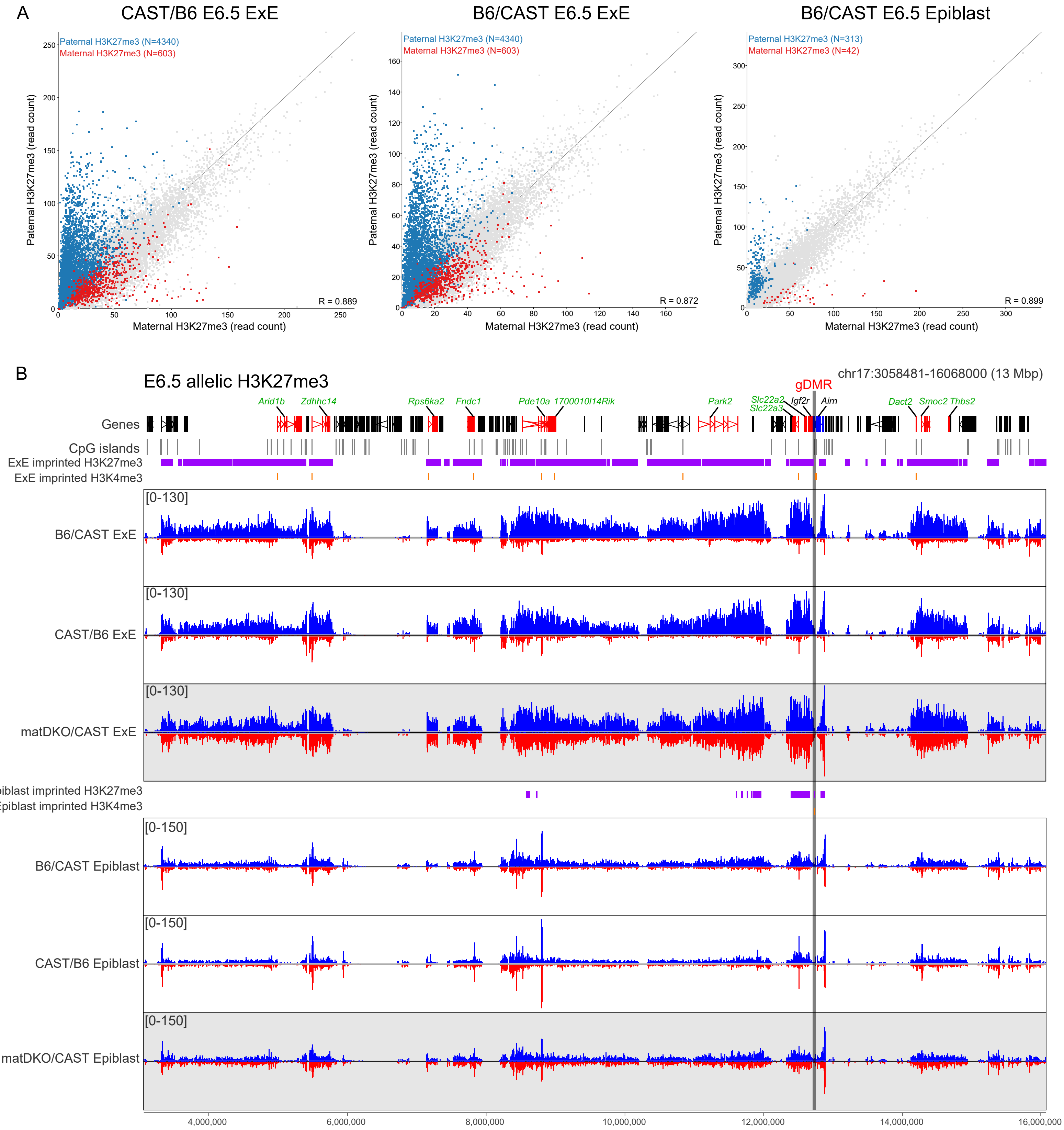

Supplementary Figure 10

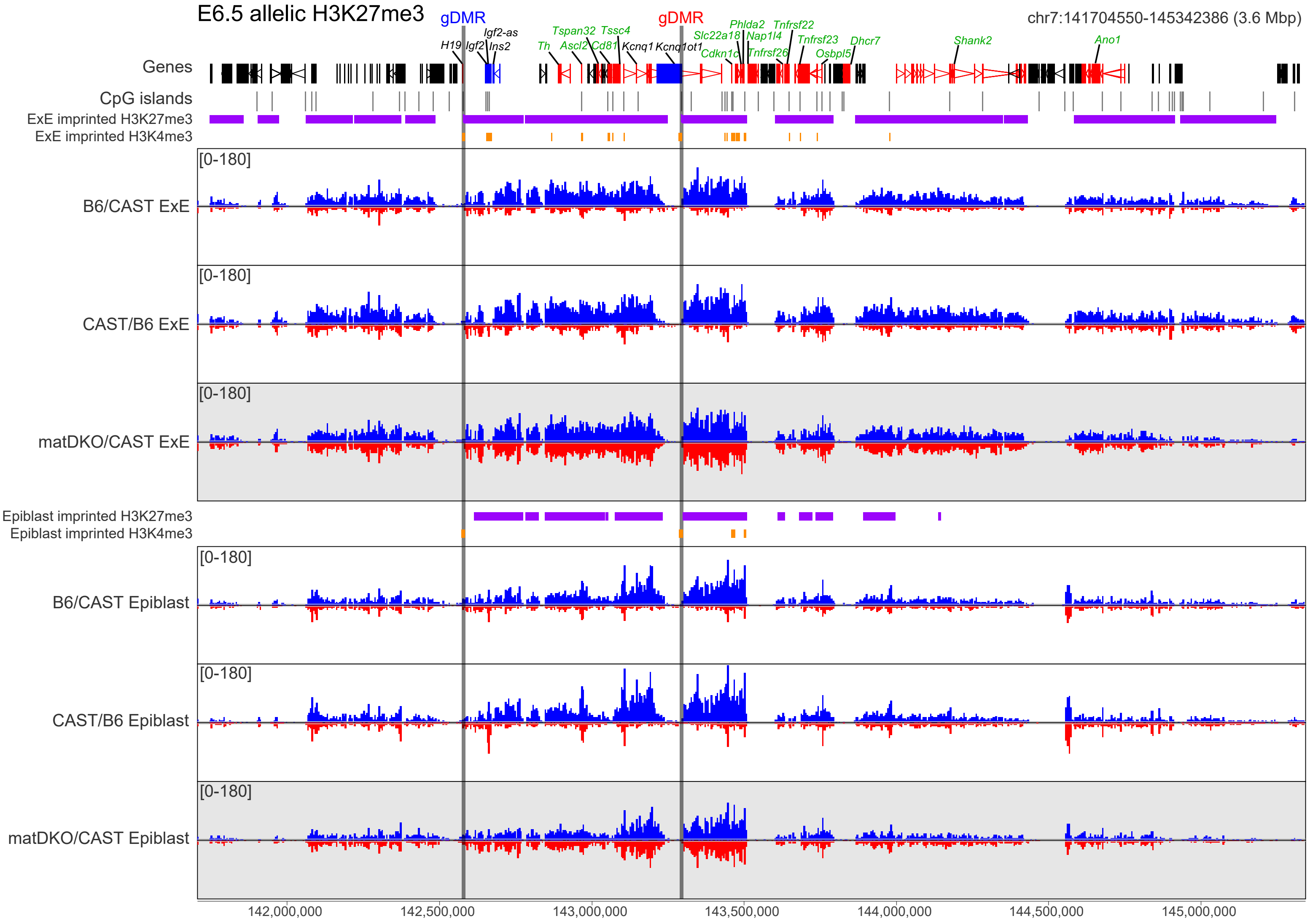

Supplementary Figure 11

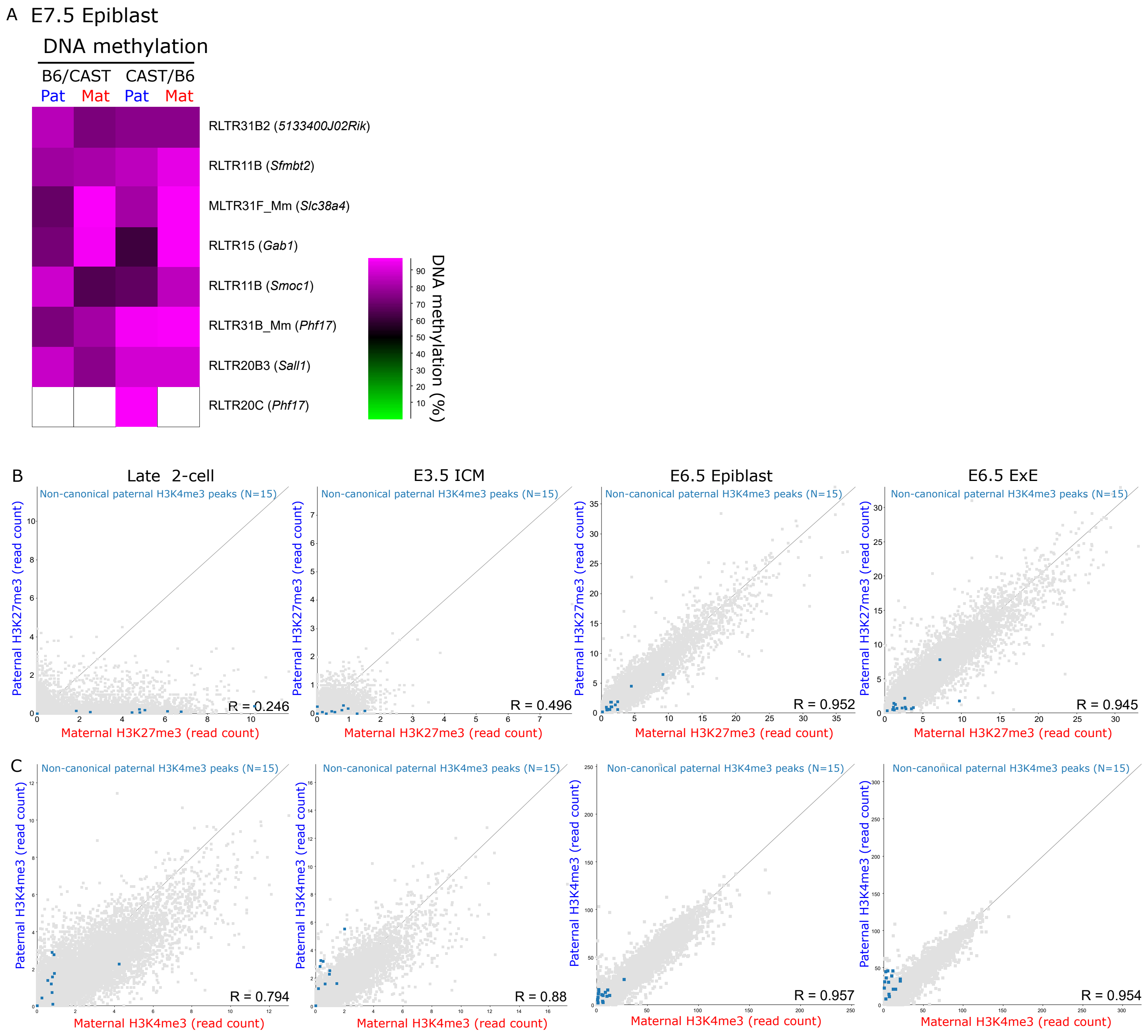

Supplement: Supplementary file 1 — Additional file 1. Supplementary figure and table legends, Figures. S1-S11. [file 13059_2019_1833_MOESM1_ESM.pdf]
