# Supplementary material for: Endogenous retroviral insertions drive non-canonical imprinting in extra-embryonic tissues
Source: Genome Biol. 2019 Oct 29;20:225. doi: 10.1186/s13059-019-1833-x (PMC6819472; doi:10.1186/s13059-019-1833-x)
Supplement: Supplementary file 3 — Additional file 3. Review history. [file 13059_2019_1833_MOESM3_ESM.docx]

Review History

**First round of review**

**Reviewer 1**

**Are you able to assess all statistics in the manuscript, including the appropriateness of statistical tests used?**

No.

**Comments to author:**

In this manuscript, Hanna and colleagues study imprinted gene regulation in the epiblast and extraembryonic ectoderm (ExE) of the mouse E6.5 embryo. Their study relies on the allelic characterization of histone marks for active promoters (H3K4me3), transcriptional elongation (H3K36me3) and transcriptional repression (H3K27me3), DNA methylation and gene expression by using reciprocal crosses of hybrid wild-type embryos, as well as DNA methylation maternally-depleted embryos. To this aim, they performed sophisticated techniques such as ultra low-input ChIP, PBAT and RNA-seq of limited number of post-implantation embryo cells.
Using these datasets, they went on:
- 1) Identifying imprinted regions in post-implantation epiblast and ExE by allelic H3K4me3 ChIP-seq peak calling. Those regions are highly predictive of mono-allelic expression, in conjunction with allelic H3K36me3 peaks and gene expression analysis; 
- 2) Describing a role for bivalent chromatin in poising the expression of some imprinted genes during placenta development 
- 3) Describing the allelic H3K27me3 enrichment at the Igf2r/Airn and Kcnq1/Kcnq1ot1 imprinting clusters in ExE and its dependence towards maternal DNA methylation, 
- 4) Reporting the existence of new, non-canonical imprints (independent of DNA methylation), through the study of allelic H3K4me3 peaks in DNA methylation maternally-depleted embryos. Interestingly, those genes, particularly in ExE, mostly coincide with transcriptionally active ERV LTRs
- 5) Dissecting the multi-layered regulation of both canonical and non-canonical regulation at the Slc38a4 imprinted locus. 
- 6) Describing the non-maintenance of oocyte-inherited H3K27me3 marks beyond preimplantation development confirming the former work by Inoue et al (2017) and indicating that non-canonical imprinting in ExE cannot rely on maternally inherited H3K27me3, but may rather result from continuous transcription from the parental allele that inherits an H3K27me3-free status.  
Although the work is partially redundant with what was previously published by Inoue and colleagues, it significantly widens our knowledge of imprinted gene regulation in extraembryonic tissues and, importantly, points towards potential regulatory mechanisms of non-canonical genomic imprinting. It brings interesting ideas, such as the requirement of other repressive marks in the control of non-canonical imprinting (other than DNA methylation and/or H3K27me3), and the potential co-option of ERV LTRs in the imprinted regulation in extraembryonic tissues. 
As such, the authors provide  here an interesting characterization and a useful resource for future studies. However the manuscript suffers from a clear lead, and this is obvious as soon as reading the abstract. It is difficult to extract what is the main message, there are several conclusions, interesting per se, but none of them are thoroughly followed, giving a feeling of superficiality. Some descriptions should be discarded, while the most interesting and novel insights should be further followed, to go beyond correlations. I suggest below  several points to be improved: 

Major Points:
Structure: 
-     In my opinion, the main message of the manuscript relates to the identification of new canonical and non-canonical imprinted genes in ExE (mainly) based on scoring for parent-of-origin enrichment of H3K4me3 (not H3k27me3), and the allelic expression of overlapping/nearby genes. Interestingly the authors identify the presence of ERV LTRs nearby the newly characterized non-canonical imprinted loci, pointing towards potential involvement in their emergence and/or regulation. I consider this is the main novelty of the paper, but genetic proof is still lacking. Conversely, although the authors investigate the function of H3K27me3 in genomic imprinting, I do not think that those results are the main stress of the paper as the title suggests. Therefore, I suggest a change in the structure of the paper.

Results:
-     Section "Imprinted bivalent chromatin poising genes" (sentences 145 - 159). Even though the idea of poised imprinted genes in the placenta is really interesting, there is the possibility of a confounding effect due to an heterogeneous histone profile of a population of cells. It might be a point to include in the discussion, or to temper the language in relation to that statement.
-     Section "Non-canonical vs canonical imprinted gene regulation" (sentences 176 - 206): This part is the most interesting and novel part of the paper. For this reason I think further work is required: 1) Prove genetically that ERV LTRs are involved in the regulation of non-canonical imprinted genes by generating an ERV LTR deletion. 2) Are those non-canonical imprinted related ERV LTRs specifically expressed in the placenta? And are they expressed in the preimplantation trophectoderm ( supporting the model put forward by the authors, whereby continuous transcription is enough to regulate allelic expression even when maternal H3K27me3 disappears?) Could you provide this information?. 3) What is their DNA methylation status (allelic) in the ExE? This should at least been shown for the Slc38a4 gene.
-     Section "Non-canonical imprinted regions lose oocyte-derived H3K27me3 during embryogenesis" (sentences 240 - 263). 1) It is not clear how many of the non-canonical imprinted genes (defined by H3K4me3) show an allelic enrichment for H3K27me3 at ExE, please provide a list or informative analysis. 2) Maybe it would be informative to include a graph that represents the H3K27me3 levels +/- error bars of oocytes, preimplantation development (publically available data), and epiblast and ExE (this work's data).
-     About the canonical and non-canonical regulation of the Slc38a4 imprinted gene. Maternal DNA methylation-dependent regulation is important in E6.5 epiblast, but dispensable in ExE. What about the status of regulation prior to implantation then? In the blastocyst (provided that Slc38a4 is significantly expressed at this stage). 
Discussion:
-     Sentences 267 - 270: This sentence sounds a bit overstated. The results of the manuscript do not unveil H3K27me3 functional roles.
-     Sentence 282 - 284: Cite Inoue et al. These are similar conclusions.

Minor points:
Background: 
-     Even though interesting and well-written, some parts of the background are not required for the comprehension of the manuscript. If required for space, background should be revisited and shortened. As examples: "mechanisms regulating the protection of DNA methylation at imprinted gDMRs" (sentences 69-72) or the part related to "parental conflict theory" (sentences 79 - 81).
Results:
-     Sentence 110: The number of cells is 2000 (also in sentence 659, Fig1 legends), but the estimated number of cells is 2500 in the methods section (sentence 374). Please verify or clarify.
-     Sentence 232-233: the authors are referring to LTR initiated transcription. Include here citations of Veselovska L. (2015) Genome Biol and Brind'amour J (2018) Nat Comm?
Figures:
-     Figure 2B: It will be interesting to include gene expression levels of those genes for the different tissues.
-     Figure 4A: Are those H3K27me3 tiles imprinted (enriched on the maternal allele in reciprocal crosses)? Please provide the data for the Cast/B6 crosses.
-     Fig 4B and Suppl Fig5: It would be clearer if it is indicated in the figure that the screenshoot depicts H3K27me3.
-     Legend Fig 2 (sentence 681): Please define the mouse strain "F".

**Reviewer 2**

**Are you able to assess all statistics in the manuscript, including the appropriateness of statistical tests used?**

No.

**Comments to author:**

This study by Hanna et al. investigated whether histone modifications play roles in regulating imprinted gene expression in early embryonic and extra-embryonic tissues. They provided a comprehensive set of data and analyses in reciprocal hybrid embryos and DNA methylation deficient embryos. Taking advantage of these datasets, the authors made a number of interesting findings including 1) Identified DNA methylation dependent (canonical) and independent (non-canonical) imprints in embryonic and extraembryonic tissues; 2) Non-canonical imprints are preferentially located at ERV LTR repeat regions and function as alternative promoters in ExE; 2) Extended the role of histone bivalency in regulating imprinting gene expression; 4) Demonstrated non-canonical H3K27me3 imprints from oocytes are not maintained in postimplantation development including extraembryonic tissues. I believe that these are important datasets that will be of benefit to the field. Therefore, I would be positive for its publication if the authors could address the following questions.

Major comments:

1.     It is interesting that many non-canonical imprints are associated with ERVs. Given they are also correlated with oocyte H3K27me3 (despite the fact that such H3K27me3 is dispensable for its maintenance), is there a link between oocyte H3K27me3 and these ERVs? In another word, what is the molecular mechanism underlying the allele-specific expression of ERVs specifically in extraembryonic tissues?        

2.     A related question is do non-canonical H3K4me3 imprints emerge in ExE? Are they inherited from pre-implantation embryos or newly established in ExE? It would be helpful if the authors can use published datasets to explore the dynamics of these non-canonical imprints during development.

3.     Do these imprinted ERV sequences have any significant motif enrichment?

4.     The author stated 87% of non-canonical imprinted genes have H3K27me3 in oocytes. What about the rest? Presumably they are imprinted through neither DNA methylation nor H3K27me3. It should be interesting to examine these genes. 

5.     For the Igf2r/Airn imprinting clusters, the author found the establishment of paternal specific H3K27me3 domain (12Mb) near this region in ExE is entirely dependent on the maternal gDMR. In the model, the author proposed that the lncRNA may play a role in the paternal H3K27me3 cluster formation similar as Xist. I believe it would be very interesting if the authors can show evidence for the existence of such lncRNA. Can the authors examine the RNA-seq data and see if this is indeed the case? 

6.     Page 9, I think it is interesting but not fully convincing. As the epiblast also shows allelic H3K4me3 peaks in these regions (although a bit lower), there is no direct evidence showing that the allelic expression of Slc38a4 is due to the allelic expression of the 'Non-canonical imprinted paternal ERVK LTR'.


Minor comments:

7.     Line 126, ExE (N=914) should be N=913.
8.     The stage information (embryonic day) is missing in some figures. 
9.     In Figure S2A, why does this PCA analysis show a significant difference between CAST/B6 and B6/CAST embryos? Did the author remove SNPs when calculating the methylation levels for all embryos?

**Authors Response**

**Point-by-point responses to the reviewers’ comments:**

Comment

*Response*

*We have sought to address all of the reviewers’ comments and, as such, made extensive changes to the manuscript to refocus on the key novel finding – the non-canonical imprinting of ERVK LTRs. Briefly, the major changes to the manuscript include: 
- We reduced the focus on the role of H3K27me3, by either removing (Figures 3, 7A, 7B, 8) or moving figures to supplemental (Figures 4, 6, 7C) from the original manuscript. 
- Building off of the identification of non-canonical imprints (Figure 3), we further explored: the expression patterning of imprinted ERVK LTRs, their sequence composition and epigenetic patterning in oocytes, the role of the imprinted ERVK LTR at the Gab1 locus, and the epigenetic regulation of non-canonical imprints in pre- and post-implantation embryos. 
• These changes included additional analyses for new figures (Figs 4-7) and revising the results, discussion, conclusions, abstract and title. 
• These changes also required two key additional experiments: 
1) CRISPR-targeting of the non-canonically imprinted ERVK LTR promoter at the Gab1 locus in hybrid embryos and generating RNA-seq data to evaluate allelic expression 
2) generating PBAT libraries from E7.5 ExE and epiblast from reciprocal hybrids for deep sequencing to evaluate allelic DNA methylation across non-canonically imprinted ERVK LTR promoters 

We have provided a point-by-point response below. Responses are provided in bullet points.* 

Reviewer 1 
In my opinion, the main message of the manuscript relates to the identification of new canonical and non-canonical imprinted genes in ExE (mainly) based on scoring for parent-of-origin enrichment of H3K4me3 (not H3K27me3), and the allelic expression of overlapping/nearby genes. Interestingly the authors identify the presence of ERV LTRs nearby the newly characterized non-canonical imprinted loci, pointing towards potential involvement in their emergence and/or regulation. I consider this is the main novelty of the paper, but genetic proof is still lacking. Conversely, although the authors investigate the function of H3K27me3 in genomic imprinting, I do not think that those results are the main stress of the paper as the title suggests. Therefore, I suggest a change in the structure of the paper. 
*• We have extensively revised the focus and structure of the manuscript to explore the genomic features and epigenetic regulation of non-canonically imprinted ERVK LTRs. We have also amended the title to represent these novel findings.* 

Results: Section "Imprinted bivalent chromatin poising genes" (sentences 145 - 159). Even though the idea of poised imprinted genes in the placenta is really interesting, there is the possibility of a confounding effect due to an heterogeneous histone profile of a population of cells. It might be a point to include in the discussion, or to temper the language in relation to that statement. 
*• We agree with the reviewer’s suggestion of possible confounds and in light of the revised focus of the manuscript, we have removed the figure on bivalency (Figure 3) and have just included this point in the discussion (lines 333-338).* 

Results: Section "Non-canonical vs canonical imprinted gene regulation" (sentences 176 - 206): This part is the most interesting and novel part of the paper. For this reason I think further work is required: 
1) Prove genetically that ERV LTRs are involved in the regulation of non-canonical imprinted genes by generating an ERV LTR deletion. 
*• We have undertaken in vivo targeting of the Gab1 ERVK LTR using CRISPR/Cas9 in hybrid embryos. We were able to excise the Gab1 ERVK LTR on the paternal allele in a single embryo (1/42 collected embryos) and generated RNA-seq data from embryonic and extra-embryonic tissues (placenta and yolk sac) from E12.5 WT (N=3) and KO (N=1) embryos. While our deletion was mosaic, meaning the effect is not completely penetrant, we do observe significant loss of imprinting in both extra-embryonic tissues. These additional experiments are included in Figure 5F and Supplementary Figure 7, results (lines 205-213) and methods (lines 367-392). We have also added additional analyses of an allelic comparison of intron-spanning reads initiating from the Gab1 ERVK LTR and exon 1 of extra-embryonic and embryonic tissues, demonstrating imprinted gene expression only initiates from the ERVK LTR promoter (Figure 5E).* 

2) Are those non-canonical imprinted related ERV LTRs specifically expressed in the placenta? And are they expressed in the preimplantation trophectoderm (supporting the model put forward by the authors, whereby continuous transcription is enough to regulate allelic expression even when maternal H3K27me3 disappears?) Could you provide this information? 
*• We have included additional analyses of publically available data, which has demonstrated that the non-canonical ERVK LTRs are specifically expressed in extra-embryonic tissues in later development (E12.5), but in the pre-implantation embryo they are expressed in both embryonic and extra-embryonic lineages. We have included these analyses in Figures 3D and 6C, respectively. 
We have expanded our investigation into the epigenetic regulation of non-canonical imprinted ERVK LTRs, and have thus revised our discussion of potential mechanisms (detailed below).* 

3) What is their DNA methylation status (allelic) in the ExE? This should at least been shown for the Slc38a4 gene. 
*• We agree that this is an important point, but we were unable to do this analysis effectively with the coverage we were able to obtain from our PBAT data from 10% input samples from E6.5 hybrid embryos. Thus, we prepared PBAT libraries from epiblast and ExE from reciprocal E7.5 hybrid embryos, where the abundance of material allowed us to deeply sequence these PBAT libraries (>100 million reads per replicate) using longer reads (150bp paired-end sequencing). These additional data revealed that non-canonical imprinted ERVKs in fact become tissue-specific secondary imprinted DMRs (ie. DNA methylation was acquired on the maternal allele in ExE, whereas, in the epiblast the LTRs became bi-allelically silenced by DNA methylation). These data and analyses have been added to the manuscript in Supplementary Table 1, Figure 6A, Supplementary Figure 11, results (lines 261-270) and methods (lines 440-445).* 

Results: Section "Non-canonical imprinted regions lose oocyte-derived H3K27me3 during embryogenesis" (sentences 240 - 263). 1) It is not clear how many of the non-canonical imprinted genes (defined by H3K4me3) show an allelic enrichment for H3K27me3 at ExE, please provide a list or informative analysis. 2) Maybe it would be informative to include a graph that represents the H3K27me3 levels +/- error bars of oocytes, preimplantation development (publically available data), and epiblast and ExE (this work's data). 
*• Unfortunately, it is challenging to quantitatively compare the levels of H3K27me3 across embryogenesis, as the genome-wide levels and distribution of H3K27me3 are changing dramatically. Thus, the best way we found to demonstrate the abundance and allelic bias of non-canonical imprints was their position within the distribution of data within each timepoint in development, as provided in Figure 7C (now Supplementary Figure 11). However, to improve clarity we have now added a heatmap of allelic enrichment at non-canonical imprinted ERVK LTRs for H3K27me3 and H3K4me3 to demonstrate the degree of enrichment on both alleles in E6.5 ExE (Figure 6A). We have also reported how many non-canonical imprints fall within an imprinted H3K27me3 peak in ExE (N=1) in the results (lines 254-257).* 

Results: About the canonical and non-canonical regulation of the Slc38a4 imprinted gene. Maternal DNA methylation-dependent regulation is important in E6.5 epiblast, but dispensable in ExE. What about the status of regulation prior to implantation then? In the blastocyst (provided that Slc38a4 is significantly expressed at this stage). 
*• We have included the expression and DNA methylation status of all non-canonical imprinted ERVK LTRs across pre-implantation development using publically available C57BL/6 data in Figure 6B and 6C.* 

Discussion: Sentences 267 - 270: This sentence sounds a bit overstated. The results of the manuscript do not unveil H3K27me3 functional roles. 
*• We have revised the entire discussion section to highlight the aspects of novelty of the revised manuscript.* 

Discussion: Sentence 282 - 284: Cite Inoue et al. These are similar conclusions. 
*• The conclusions have been revised to reflect the novel findings of the revised manuscript.* 

Background: Even though interesting and well-written, some parts of the background are not required for the comprehension of the manuscript. If required for space, background should be revisited and shortened. As examples: "mechanisms regulating the protection of DNA methylation at imprinted gDMRs" (sent 69-72) or the part related to "parental conflict theory" (sent 79 - 81). 
*• We agree that these points are tangential and have removed them.* 

Results: Sentence 110: The number of cells is 2000 (also in sentence 659, Fig1 legends), but the estimated number of cells is 2500 in the methods section (sentence 374). Please verify or clarify. 
*• We have clarified this point to be approximately 2500 throughout the manuscript, it was estimated based on the expectation that 660 cells are in an E6.5 epiblast (Snow. 1977 J Emryol Exp Morphol;42:293-303.).* 

Results: Sentence 232-233: the authors are referring to LTR initiated transcription. Include here citations of Veselovska L. (2015) Genome Biol and Brind'amour J (2018) Nat Comm? 
*• These citations have been added (line 242).* 

Figure 2B: It will be interesting to include gene expression levels of those genes for the different tissues. 
*• We have provided the expression levels of non-canonical imprinted ERVK LTRs across tissues in post-implantation development; include E7.5, E12.5, and E16.5 (Figure 3D).* 

Figure 4A: Are those H3K27me3 tiles imprinted (enriched on the maternal allele in reciprocal crosses)? Please provide the data for the Cast/B6 crosses. 
*• We have added the scatterplot for the CAST/B6 cross (now in Supplementary Figure 9A).* 

Fig 4B and Suppl Fig5: It would be clearer if it is indicated in the figure that the screenshoot depicts H3K27me3. 
*• These figures have been amended (now Supplementary Figures 9 and 10).* 

Legend Fig 2 (sentence 681): Please define the mouse strain "F". 
*• F denotes FvB, this has been added to the Figure legend.* 

Reviewer 2 
1. It is interesting that many non-canonical imprints are associated with ERVs. Given they are also correlated with oocyte H3K27me3 (despite the fact that such H3K27me3 is dispensable for its maintenance), is there a link between oocyte H3K27me3 and these ERVs? In another word, what is the molecular mechanism underlying the allele-specific expression of ERVs specifically in extraembryonic tissues? 
*• We sought to address the question “why are these LTRs imprinted and not others that are also expressed?” Therefore, we identified a subset of ERVK LTRs that were expressed in extra-embryonic tissues, but which were not imprinted (methods, lines 516-527, Supplementary Table 6). These ‘active ERVK LTRs’ were predominantly marked by H3K4me3 in the oocyte, in contrast to non-canonical imprinted ERVKs which were marked by H3K27me3. Both were significantly different than a random subset of inactive ERVK LTRs, demonstrating that the epigenetic status inherited from the oocyte at these repetitive elements plays a role in their transcriptional regulation in extra-embryonic tissues (Figure 4C).* 

2. A related question is do non-canonical H3K4me3 imprints emerge in ExE? Are they inherited from pre-implantation embryos or newly established in ExE? It would be helpful if the authors can use published datasets to explore the dynamics of these non-canonical imprints during development. 
*• We have included the analysis of allelic H3K4me3 across pre-implantation development to Supplementary Figure 11C.* 

3. Do these imprinted ERV sequences have any significant motif enrichment? 
*• We investigated whether non-canonically imprinted ERVKs and ERVK promoters active in extra-embryonic tissues were enriched motifs in both an unbiased analysis and specifically evaluating trophoblast TFs: CDX2, EOMES and ELF5 (methods, lines 528-532). We were unable to find a motif enrichment that explained their expression pattern (results, lines 181-185); however, we found that these ERVK LTRs were remarkable in their high CpG content and proximity and strandedness to TSSs. These results have been added in Figures 4A and 4B.* 

4. The author stated 87% of non-canonical imprinted genes have H3K27me3 in oocytes. What about the rest? Presumably they are imprinted through neither DNA methylation nor H3K27me3. It should be interesting to examine these genes. 
*• We have revised this analysis to specifically investigate the epigenetic status of transcribed non-canonically imprinted ERVK LTRs rather than the associated imprinted genes, which allowed us to compare the epigenetic regulation of imprinted ERVK LTRs to active and inactive ERVK LTRs (Figure 4C).* 

5. For the Igf2r/Airn imprinting clusters, the author found the establishment of paternal specific H3K27me3 domain (12Mb) near this region in ExE is entirely dependent on the maternal gDMR. In the model, the author proposed that the lncRNA may play a role in the paternal H3K27me3 cluster formation similar as Xist. I believe it would be very interesting if the authors can show evidence for the existence of such lncRNA. Can the authors examine the RNA-seq data and see if this is indeed the case? 
*• The activity and necessity of the lncRNA in the imprinting of the Igf2r/Airn has been demonstrated in cultured trophoblast stem cells by other groups, including a very recent study by Schertzer et al. 2019 Mol Cell. We have revised our discussion to clarify this point and added this recent citation (lines 341-344).* 

6. Page 9, I think it is interesting but not fully convincing. As the epiblast also shows allelic H3K4me3 peaks in these regions (although a bit lower), there is no direct evidence showing that the allelic expression of Slc38a4 is due to the allelic expression of the 'Non-canonical imprinted paternal ERVK LTR'. 
*• We agree that we have over-interpreted this finding without further genetic evidence, and have amended our results section to highlight it as one potential mechanism which may be regulating Slc38a4 (lines 244-249).* 

7. Line 126, ExE (N=914) should be N=913. 
*• This has been corrected.* 

8. The stage information (embryonic day) is missing in some figures. 
*• This has been added to all appropriate figures.* 

9. In Figure S2A, why does this PCA analysis show a significant difference between CAST/B6 and B6/CAST embryos? Did the author remove SNPs when calculating the methylation levels for all embryos? 
*• The SNPs have been N-masked for the initial mapping of these datasets, so this is unlikely to be due to SNPs. We attribute this to subtle differences in the developmental timing of embryos collected from CAST females compared to B6 females, as the CAST/B6 embryos are consistently more methylated than B6/CAST despite both being collected at E6.5.*

**Second round of review**

**Reviewer 2**

In the revised paper, the authors further demonstrated that non-canonical imprints at extra-embryonic tissue are marked by H3K27me3 in oocytes, and then replaced by DNA methylation on the maternal allele, despite the fact that the regulatory mechanism remains elusive. A minor suggestion is to add the pre-implantation data in Figure 6A which would very informative to the readers. Overall, I think this revised manuscript is much improved and meet the publication requirement of Genome Biology.

**Authors Response**

**Point-by-point responses to the reviewers’ comments:**

Comment

A minor suggestion is to add the pre-implantation data in Figure 6A which would very informative to the readers.

*Response*

- *We have added a figure showing allelic H3K4me3 and H3K27me3 throughout pre-implantation development as Figure 6D.*
